# Nanobiotechnology in crop stress management: an overview of novel applications

**DOI:** 10.1186/s11671-023-03845-1

**Published:** 2023-05-15

**Authors:** Ahmad Nawaz, Hafeez ur Rehman, Muhammad Usman, Abdul Wakeel, Muhammad Shafiq Shahid, Sardar Alam, Muhammad Sanaullah, Muhammad Atiq, Muhammad Farooq

**Affiliations:** 1 Department of Entomology, University of Agriculture, Faisalabad, 38040 Pakistan; 2 Department of Agronomy, University of Agriculture, Faisalabad, 38040 Pakistan; 3 PEIE Research Chair for the Development of Industrial Estates and Free Zones, Center for Environmental Studies and Research, Sultan Qaboos University, Al-Khoud 123, Muscat Oman; 4 Institute of Soil and Environmental Sciences, University of Agriculture, Faisalabad, 38040 Pakistan; 5 Department of Plant Pathology, University of Agriculture, Faisalabad, 38040 Pakistan; 6 Department of Plant Sciences, College of Agricultural and Marine Sciences, Sultan Qaboos University, Al-Khoud 123, Muscat Oman

**Keywords:** Nanobiotechnology, Plant stresses, Abiotic factors, Biotic factors, Insect pests, Plant diseases, Nanoparticles

## Abstract

Agricultural crops are subject to a variety of biotic and abiotic stresses that adversely affect growth and reduce the yield of crop plantss. Traditional crop stress management approaches are not capable of fulfilling the food demand of the human population which is projected to reach 10 billion by 2050. Nanobiotechnology is the application of nanotechnology in biological fields and has emerged as a sustainable approach to enhancing agricultural productivity by alleviating various plant stresses. This article reviews innovations in nanobiotechnology and its role in promoting plant growth and enhancing plant resistance/tolerance against biotic and abiotic stresses and the underlying mechanisms. Nanoparticles, synthesized through various approaches (physical, chemical and biological), induce plant resistance against these stresses by strengthening the physical barriers, improving plant photosynthesis and activating plant defense mechanisms. The nanoparticles can also upregulate the expression of stress-related genes by increasing anti-stress compounds and activating the expression of defense-related genes. The unique physico-chemical characteristics of nanoparticles enhance biochemical activity and effectiveness to cause diverse impacts on plants. Molecular mechanisms of nanobiotechnology-induced tolerance to abiotic and biotic stresses have also been highlighted. Further research is needed on efficient synthesis methods, optimization of nanoparticle dosages, application techniques and integration with other technologies, and a better understanding of their fate in agricultural systems.

## Introduction

Crop production has been stagnant during the last decades while food demand is increasing sharply due to ever increasing human population [1]. It has been reported that almost 800 million people are chronically hungry and 2 billion suffer micronutrient deficiencies while 653 million people would still be undernourished in 2030 [2]. Therefore, food security will remain a huge challenge as the world’s human population will reach around 10 billion in 2050 [3]. The global losses of one-third of food produced have been estimated by FAO (Food and Agriculture Organization) in 2011 which amounts to about 1.3 billion tons per year [4]. The pre-harvest crop losses reported are around 35% due to different factors (diseases, animal pests, weeds, abiotic stresses) which account for 1051.5 Mt (million tonnes). In addition, the losses during harvesting and storage are about 690 Mt [5].

Both biotic and abiotic stresses cause significant crop yield losses. However, the global crop yield losses due to biotic stresses vary among major crops and regions [6]. Insect pests cause 15–20% yield losses in principal food and cash crops [7]. Similarly, the global estimates of yield losses due to pathogenic disease range from 11 to 30% [6, 8, 9]. Abiotic stresses (drought, water logging, temperatures, salinity, heavy metals and mineral toxicity, etc.) lead to morphological, physiological, biochemical, and molecular changes in plants that adversely affect their growth, development, and productivity [10]. They can cause significant losses (50–70%) in growth and yield [11–15]. The overproduction of reactive oxygen species (ROS) is one of the major reasons for crop losses caused by abiotic stresses [16]. The global models have predicted an increase in CO_2_ levels from 400 to 800 ppm [17, 18]. Moreover, more than 45% of arable lands are endangered by drought [19, 20], above 27% of the global area is under aridity, and most crop species are sensitive to salt stress (1.0–1.8 dS m^−1^). These abiotic stresses can cause 10 to 50% yield loss [21, 22]. In addition, heavy metal (Cr, Cd, As, Pb, Cu, Hg) pollution negatively affects seed germination, photosynthesis, respiration, and transpiration and ultimately reduces growth, yield as well as yield quality [23, 24].

The use of synthetic chemicals had been the main focus to mitigate the effects of abiotic and biotic stresses in crop plants. The estimated increase in pesticide use was up to 3.5 Mt in 2020 [25] with an estimated value of US$ 103.5 billion. The global pesticide market is predicted to reach more than US$ 107.5 to 184 billion in 2023 to 2033 respectively with a steady growth of 5.5% (https://www.persistencemarketresearch.com/market-research/pesticides-market.asp: data retrieved on 12-02-2023). The facts about health hazards and impacts on non-target organisms of pesticides revealed a 35% decrease in soil respiration, a 90% water pollution of agricultural lands, a 70% decline in insect biomass, a 50% decline in farmland birds, a 30% decline in the honey bee population, a 42% reduction in species richness in Europe, Australia and Americas. In addition, a 25–30% increase in cancer and mental health risks and a 50% risk of leukemia, lymphoma and brain cancer is linked to pesticide exposure to children [26]. Moreover, the combined impacts of different stresses increase the complexity of plant responses. Thus, a second green revolution is needed to fulfill the food demand of the human population. Therefore, this is required to find some alternative solutions focusing on environmental sustainability and human health.

**Fig. 1:**
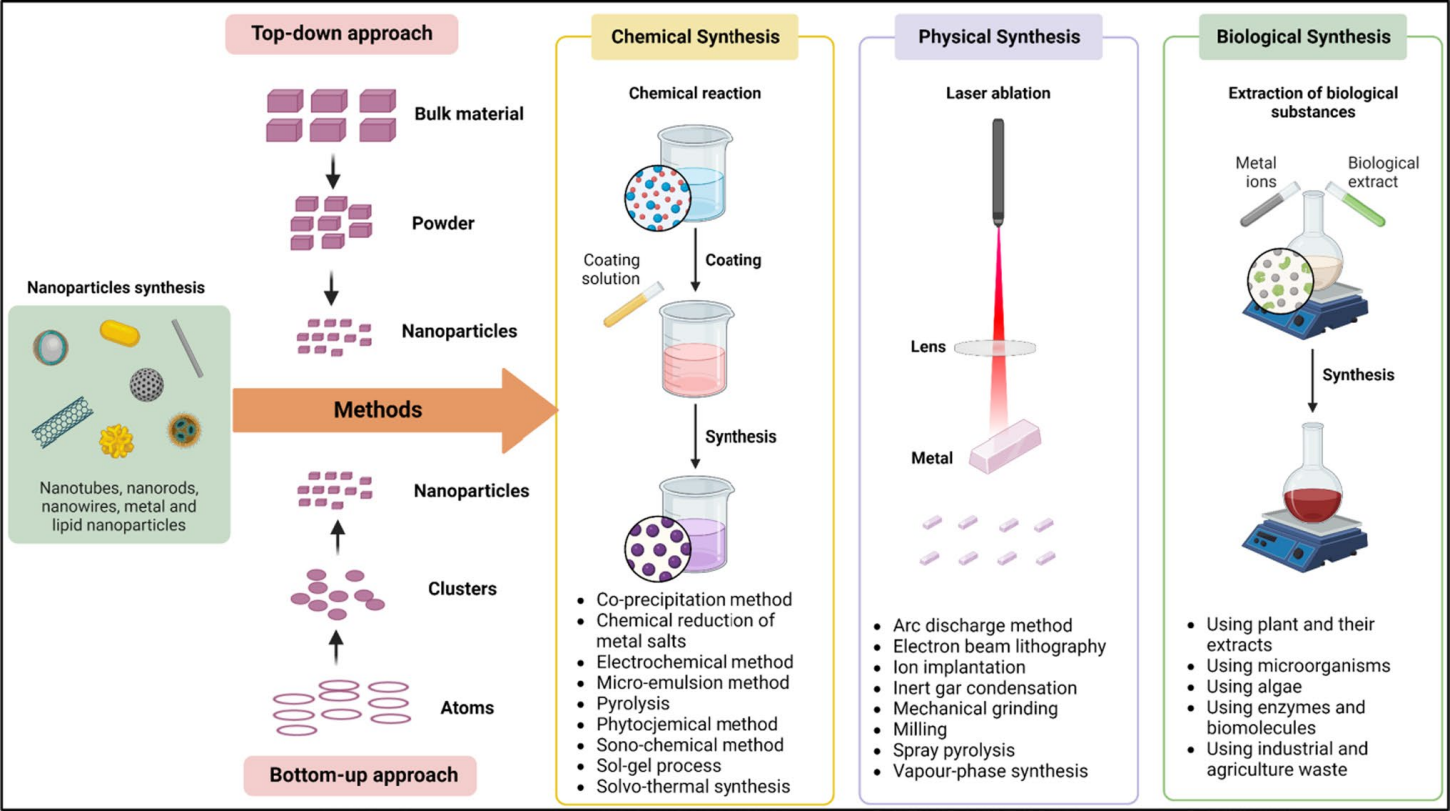
Nanoparticles synthesis with topdown and bottom up approaches showing the chemical, physical and biological synthesis methods [34]

Nanotechnology as a “Key Enabling Technology’’ [27] has the potential to serve as a key alternative to achieve the goal of sustainable agriculture [28]. Nanoparticles are synthesized through physical, chemical and biological approaches (Fig. 1) by using metal or metal oxide [29]. Metal-based nano- insecticides, pesticides, and insect repellant formulations show significant potential against plant pathogens and insect pests [30]. However, the biological synthesis (green synthesis) of nanoparticles using plants or plant extracts, entomopathogens and other biomaterial has potential benefits over other approaches. Plants or plant extracts contain enzymes, sugars, and phytochemicals like flavonoids, latex, phenolics, terpenoids, alcohols, amines and cofactors, etc. which act as reducing and stabilizing agents during the synthesis of metal nanoparticles. This helps to prepare not only the most promising and eco-friendly nanoparticles with well-defined sizes and shapes but also prevent environmental contamination [31–33]. Therefore, the present review encapsulates the innovations in nanobiotechnology applications in agriculture especially focusing on the potential of biosynthesized nanoparticles to mitigate abiotic and biotic stresses in crop production.

## Nanobiotechnology for abiotic stress tolerance

Abiotic stresses significantly affect plant growth and cause a substantial reduction in crop yield (by about 50%) [11, 13–15]. These stresses disturb the plant metabolism lead causing a significant reduction in plant growth, development, and yield formation [35]. In addition to other effects, the overproduction of reactive oxygen species (ROS) is one of the major reasons for crop losses caused by abiotic stresses [16, 36]. The application of nutrients, osmoprotectants and stress signaling compounds has been effective in scavenging the ROS and improving the tolerance against abiotic stresses [13, 15, 37]. However, the application of these materials in nano-sizes can be more effective. For example, increasing evidence indicates that delivery of the above-mentioned substances as nanomaterials improves plant tolerance against heat [38], drought [39], salinity [40] and trace metal [41] stresses. Nanomaterials have high surface energy and a high surface/volume ratio that helps improve their biochemical activity, reactivity and effectiveness when delivered to plants [28]. In plant defense, nanomaterials not only shield against ROS but also act as oxidative stress inducers at the same time [42]. The latter stimulates the plant antioxidant defense system. Novel properties of nanoparticles (NPs) exhibit their potential not only for crop management but also to deal with abiotic stresses. Several metals including silver (Ag), copper (Cu), gold (Au), iron (Fe), titanium (Ti) and zinc (Zn) and their oxides have been used to produce NPs. These NPs have been recently used for the green synthesis of NPs using plants and their extracts, micro-organisms and membranes, and DNA of viruses including diatoms. Green-synthesized NPs widely used in agriculture include AgNPs [43, 44].

In the following lines, the role of nanobiotechnology in improving tolerance against different abiotic stresses has been discussed.

### Drought stress

Drought is one of the major abiotic stresses with devastating effects on growth and productivity of crop plants [13, 45]. The US weather disaster analysis from 1980 to 2012 revealed that drought and heat stresses caused extensive agricultural losses of around $200 billion in which drought alone caused $50 billion worth of damage [46]. Drought exerts several morphological, physiological, biochemical and molecular responses in crop plants for adaptation to drought. Some adaptive mechanisms include the activation of the defence system, reducing leaf area, osmotic balance, hormonal homeostasis, expression of stress genes and shortening of plant life cycle [45]. However, only a few studies report the role of green synthesized NPs in the alleviation of drought stress. For instance, the application of green synthesized AgNPs increased antimicrobial activity against *Escherichia coli* and *Staphylococcus aureus* under drought stress in *Tephrosia apollinea* [47]. The improved response was observed with a decrease in membrane damage and an increase in hydrogen peroxide (H_2_O_2_) contents in the roots of *T*. *apollinea* under incremental drought. Likely, Ag-synthesized green NPs improved the seed germination (89.5%), germination rate (6.88) and seedling biomass in lentil under drought stress. This improvement was associated with the maintainance of tissue water balance [48]. Thus, green synthesized NPs can be used to reduce the detrimental effects of drought, however, further investigation of associated physiological, biochemical and molecular mechanisms is needed. Future studies on other metals NPs may be extended to explore their biological roles under drought.

### Salt stress

More than 20% of cultivated lands are affected salt stress. Salt stress affects the plant growth through salinity-induced osmotic stress, specific ion toxicity and mineral imbalance. The application of NPs improves plant growth, modulates carbohydrate and protein synthesis, and enhances the activities of antioxidant enzymes such as catalase (CAT), guaiacol peroxidase (POX), ascorbate peroxidase (APX) under salt stress and thus helps reducing the impact of salt stress by ROS detoxification and hormonal regulation [49]. In another study, the foliage applied Se and Cu nanofertilizer reduced the impact of saline water and significantly improved the tomato growth, nevertheless, soil properties were negatively affected due to the application of saline water [50].

**Fig. 2:**
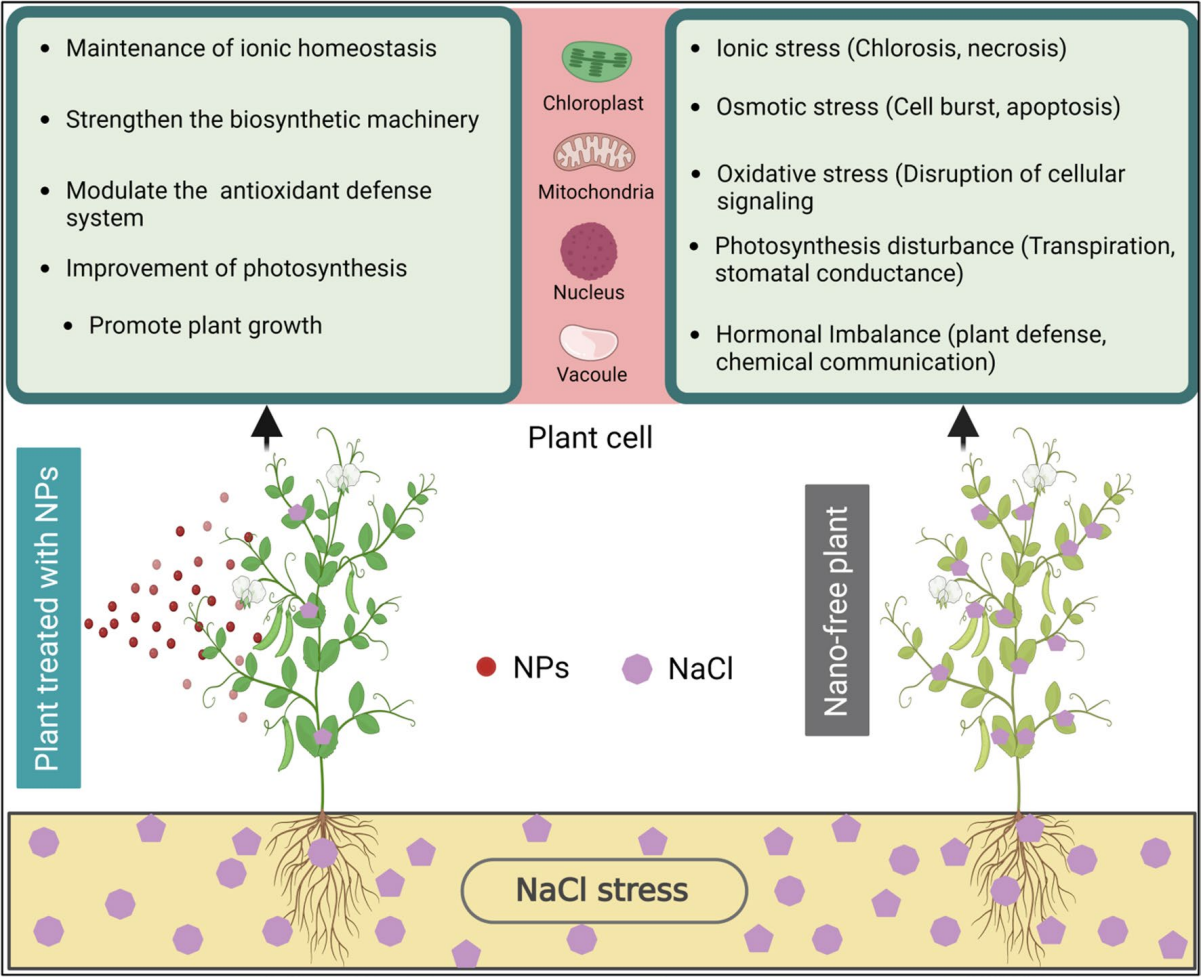
Schematic mechanisms in plant cell associated with NPs application alleviating salt stress [56]

Under salt stress, Na^+^ and Cl^−^ uptake increases that causes oxidative damage and restrict the K^+^ and Ca^2+^ uptake [51]. The Application of NPs restrict Na^+^ and Cl^−^ entry to plants under salinity. The application of ZnO-NPs improved the uptake of K^+^ and Ca^2+^ while reducing Na^+^ and Cl^−^ accumulation [52]. The NPs of iron (Fe), cesium (Cs) and cobalt (Co) play a supportive role for catalase enzyme, whereas, copper (Cu), iron (Fe), caesium (Cs) and manganese (Mn) do the same role for peroxidase (POD) enzyme [53]. The application of silver NPs at low concentrations improved antioxidant enzymatic activity and improved plant growth under salt stress conditions [54]. Likewise, the application of TiO_2_-NPs improved salt tolerance in *Dracocephalum moldavica* through the activation of antioxidant defense system that helped reduce the salinity-induced oxidative damages and improve plant growth under salt stress [55]. The use of NPs can help improve plant salt tolerance through the activation of antioxidant system, maintenance of tissue water status and ion homeostasis, etc. (Fig. 2). However, further research is desired to improve the efficiency of NPs for field-scale use to improve plant salt tolerance.

### Thermal stresses

Temperature extremes, high and low, have a strong impact on plant growth, development and yield formation [37, 57]. High temperature stress or heat stress is the rise in temperature beyond a critical threshold for a period of time sufficient to cause irreversible damage to plant growth, development, and yield [13, 58]. Heat stress induces the overproduction of ROS causing oxidative damage to plant biological membranes and other vital molecules [59, 60] and causing a significant reduction in photosynthetic pigments and carbon assimilation in wheat and chickpea [60, 61]. Low temperature may cause chilling (0–15 °C) or freezing (< 0 °C) stresses. The chilling stress causes injuries without ice crystal formation whereas the freezing stress damage plant tissues by forming ice crystals [37]. Low temperature stresses affect plant growth due to photoinhibition, ROS-induced oxidative damages, reduction in the nutrient update, activities of various enzymes and carbon assimilation [13].

The use of nanomaterials has been quite effective in improving the plant tolerance to heat stress (Table 1). For example, Iqbal et al. [62] reported significant improvement in plant biomass and leaf area in wheat under heat stress by exogenous application of AgNPs synthesized with moringa extract. The green-synthesized AgNPs balanced the tissue water content status and improved chlorophyll content under heat stress compared to the control in wheat crop [62]. The application of moringa extract-synthesized AgNPs improved the tolerance against heat stress through the activation of antioxidant defense system [62] as AgNPs application modulates the activation of antioxidant genes (MeCu/ZnSOD and MeAPX2) under thermal stresses in *Arabidopsis thaliana* [63].

**Table 1:** Role of different nanoparticles in improving tolerance against thermal stresses in different plant species

Nanoparticles (NP)	Plant species	Effects	Reference
Ag NP	Wheat	Increased the root (5 to 5.4%) and shoot length (22.2 to 26.1%), fresh (1.3 to 2%) and dry (0.36 to 0.60%) weight, and leaf area (18.3 to 33.8%) under heat stress compared to control	[62]
	Wheat	Balanced relative water content (RWC) and improved chlorophyll content under heat stress compared to control	[64]
CeO_2_ NP	Maize	Enhanced activities of catalase (2000 to 3000%) and peroxidase (162 to 1400%) compared with control, and upregulated *HSP70* expression under heat stress	[65]
Ce NP	Arabidopsis	Reduced (52%) the leaf reactive species levels, and Increased the quantum yield of photosystem II (19%), the carbon assimilation rate (67%) and the Rubisco carboxylation rate (61%) compared with control under cold and heat stresses	[38]
MWCNTs (multi-walled carbon nanotubes)	Tomato	Upregulated the expression of various stress-related genes including *HSP90* under heat stress	[66]
Na_2_SeO_4_ NP	Tomato	Improved root volume (33 to 60%) and leaf chlorophyll (19 to 18%) contents compared with control under heat and cold stresses	[67]
Se NP	Sorghum	Stimulated the antioxidant defense system by enhancing activities of antioxidant enzymes including superoxide dismutase (150%), catalase (120%) and peroxidase (40%) compared with control under heat stress	[68]
Se NP	Chrysanthemum	Improved plant biomass through the activation of antioxidant enzymes (peroxidase and catalase)	[69]
SiO_2_ NP	Wheatgrass	Overcame seed dormancy, and improved seed germination (28%) and seedling weight (49%) compared with control under cold stress	[70]
TiO_2_ NP	Tomato	Increased photosynthetic efficiency under heat stress compared with control	[71]
	Chickpea	Enhanced activities of antioxidative enzymes (superoxide dismutase, catalase, ascorbate peroxidase, glutathione peroxidase, guaiacol peroxidase, polyphenol oxidase, lipoxygenase, allenoxide synthase) and chlorophyll contents (1–50%), and decreased H_2_O_2_ content (27 to 31% and electrolyte leakage (7 to 25%) compared with control under cold stress	[72]
	Chickpea	Increased the Rubisco activity (7%), decreased the H_2_O_2_ contents (29 to 34%) compared with control under cold stress	[73]
	Chickpea	Decrease the rate of electrolyte leakage (14 to 32%) and increased the expression of cold tolerance genes compared with control under cold stress	[74]
Chitosan NP	Banana plants (*Musa acuminata* var. Baxi)	Decreased the lipid peroxidation (33%), reactive oxygen species (H_2_O_2_, ^•^OH, and O_2_^•−^ by 33, 33, 40, and 48%, respectively), and increased the fresh (14%) and dry weights (41%), and antioxidant activities (peroxidase (and superoxide dismutase by 8 and 17%, respectively), and oxidative damages under cold stress	[75]

Selenium (Se) is known for its vital role in the plant antioxidant defense system. Application SeNPs, at low concentration, stimulated the antioxidant defense system by enhancing the activities of antioxidant enzymes, and improved the photosynthetic pigments and plant growth of tomato [67] and grain sorghum [68]. The application of SeNPs, green-synthesized with a bacterial strain *Lactobacillus casei*, improved the tolerance against heat stress in chrysanthemum through the activation of the antioxidant defense system [69]. The application of titanium dioxide (TiO_2_) NPs also contributed to heat tolerance in tomato by regulating the stomatal oscillation [71].

Heat shock proteins (HSPs) are molecular chaperones expressed in plant exposure to heat stress [76]. These HSPs induce heat tolerance in plant species by stabilizing the protein structure [77]. The application of nanomaterials has been very effective in inducing heat stress through the expression of HSPs (Table 1). For instance, Zhao et al. [65] reported that the application of cerium oxide (CeO_2_) NPs improved maize growth under heat stress due to the upregulation of HSP70. In another study, the application of multi-walled carbon nanotubes (MWCNTs) improved tomato growth under heat stress through the expression of various stress-related genes including HSP90 [66].

The use of nanomaterials has been found effective to induce cold tolerance in different plant species. For example, SeNPs application improved the photosynthetic pigments, activated the plant antioxidant defense system and increased tomato plant growth under cold stress [67]. Likewise, the application of titanium oxide (TiO_2_) NPs improved the cold tolerance in chickpea through a significant increase in the activities of antioxidant enzymes (superoxide dismutase, catalase, ascorbate peroxidase, glutathione peroxidase, guaiacol peroxidase, polyphenol oxidase, lipoxygenase, allenoxide synthase), chlorophyll contents, and a significant reduction in ROS and oxidative damage compared with control under cold stress [78] The application of TiO_2_NPs also induced the expression of cold tolerance genes [74].

Plant photosynthesis, the key physiological process responsible for food production for all, is sensitive to cold stress [16]. In addition to ROS-induced oxidative damages, cold stress causes a significant reduction in photosynthetic pigments and activity of key photosynthetic enzymes including Rubisco carbon resulting in a decrease in photosynthetic rate [13, 79]. However, the application of nanomaterials has been found to reduce the ROS-induced damage in the thylakoid membrane [80, 81] and improves the light absorption capacity of chloroplast [82], electron transport rate and the activity and efficiency of Rubisco [73, 83, 84]. The application of TiO_2_ NPs in chickpea caused a significant increase in the activities of antioxidant enzymes (superoxide dismutase, catalase, ascorbate peroxidase, glutathione peroxidase, guaiacol peroxidase, polyphenol oxidase, lipoxygenase, allenoxide synthase), chlorophyll contents [78] and expression of genes controlling the chlorophyll-binding protein and Rubisco [73] under cold stress and increased plant cold tolerance (Table 1).

### Toxic metals stress

Soil contamination by toxic metals has been recognized as an important threat to plant development, soil ecosystem, and human health. Metals are grouped into two categories including (i) essential metals and (ii) non-essential metals. Essential metals are required to support plant development as micronutrients. These include cobalt (Co), copper (Cu), iron (Fe), manganese (Mn), molybdenum (Mo), nickel (Ni), selenium (Se), and zinc (Zn). Nevertheless these essential metals becomes toxic to plants if their concentration increases the threshold levels [85]. On the other hand, non-essential metals like arsenic (As), cadmium (Cd), chromium (Cr), lead (Pb), mercury (Hg), and silver (Ag),do not have any biological role in plantsand can have detrimental impacts even at low concentration [86].

Toxic metals stress affect plant metabolism by disturbing the protein structure, hampering functional groups of vital molecules, and causing oxidative damage to biological membranes [87–89].

The use of various nanomaterials with microbes simultaneously or sequentially, can impede the effects of toxic metals in plants. For example, the inoculation of arbuscular mycorrhizal fungi along with nanoscale zero-valent iron (nZVI) even in lower amounts (100 mg kg^–1^) improved the immobilization of Cd reducing their uptake in sweet sorghum in acidic polluted soil [90]. Similarly, the use of polyvinylpyrrolidone-coated iron oxide (Fe_2_O_3_) nanoparticles along with Gram-negative bacterium *Halomonas* sp. completely removed Cd and Pb while shortening the remediation time. On the other hand, nanoparticles alone removed about 66% of Cd and 82% of Pb as compared to 84% of Cd and 81% of Pb removal by bacterium alone [91].

**Fig. 3:**
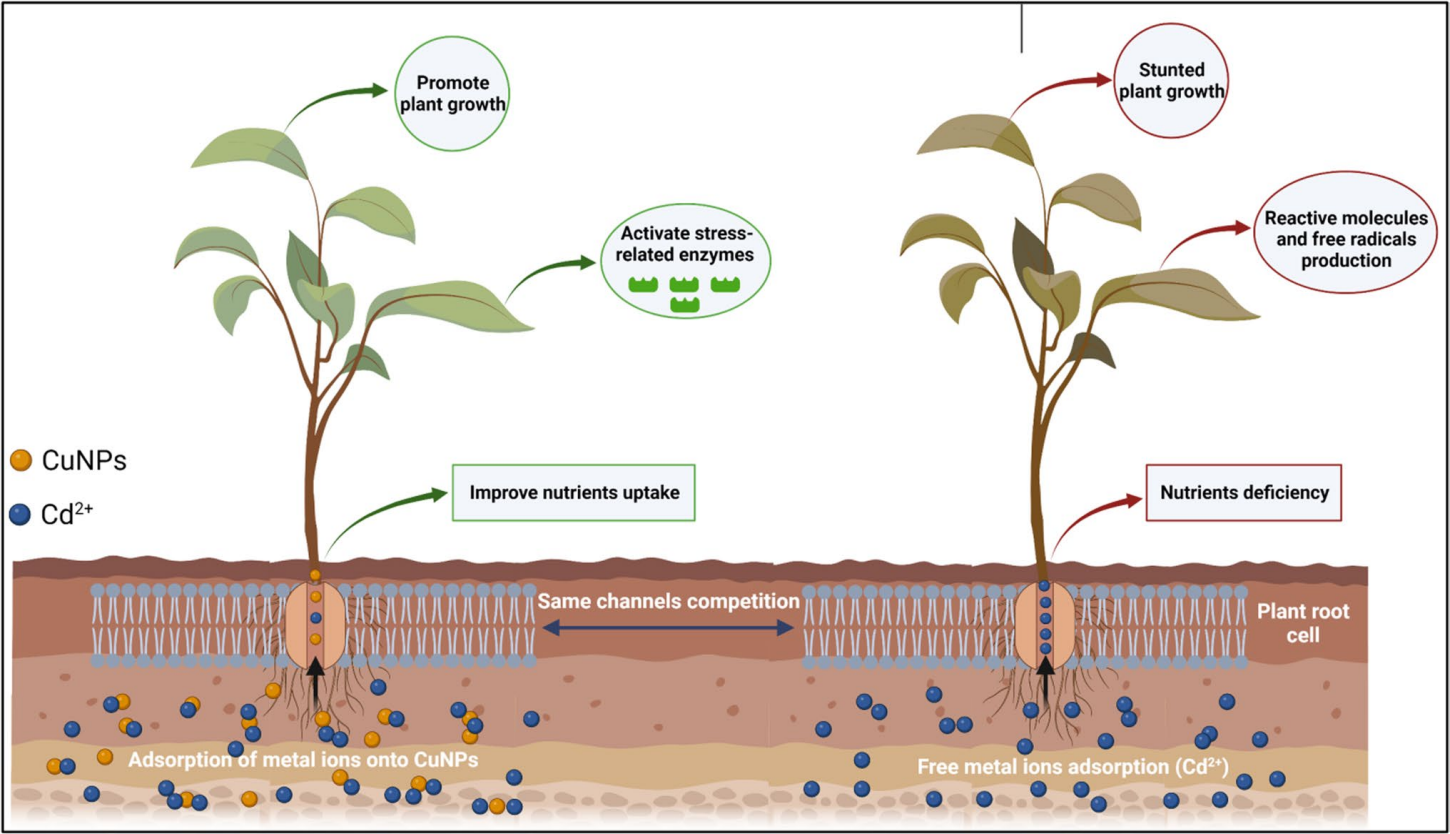
The role of biogenic copper nanoparticles (CuNPs) in reducing the translocation of Cd from soil to plants and facilitating the plant to activate defense system to combat Cd-stress. This figure is reproduced with permission from [94]

Nanomaterials can also improve the phytoextraction efficiency for soil remediation. For example, *Lolium perenne* plants in the presence of nZVI (100 mg kg^–1^) showed the highest accumulation of Pb (1175 μg per pot). However, higher doses of nZVI (2000 mg kg^–1^) reduced the uptake of Pb (to 832 μg per pot) by causing oxidative stress to plants [92]. Nanobiotechnology can also rely on the use of biologically synthesized materials to combat toxic metal stress in soils [93, 94]. For example, biogenic copper nanoparticles developed by involving a copper-resistant bacterium (*Shigella flexneri* SNT22) decreased Cd uptake (by 50%) from contaminated soil to wheat plants [94]. This treatment also improved the shoot dry weight (by 28%), plant length (by 44%), and the contents of nitrogen (41%), and phosphorus (58%). It has been proposed that biogenic copper nanoparticles adsorb Cd on its surface and prevent its uptake by plants [94]. Moreover, these nanoparticles hinder metal translocation into plants by competing with metal for membrane entry channels. After they enter into the plant body, these nanoparticles activate many defense-related enzymes reducing the metal translocation to plants (Fig. 3).

Despite the potential of nanobiotechnology in alleviating metal stress, there exist limited studies in this emerging field. The integration of nanotechnology and bioremediation can combine the benefits of both strategies and can facilitate the development of sustainable remediation technology. However, the biogeochemistry of contaminated soils can have a significant effect on the efficiency and interactions of nanoparticles with pollutants and microbes [95]. This needs to be explored for field application with an emphasis on the fate and environmental impacts of nanomaterials.

### Organic pollutants stress

Organic pollutants (OPs) are ubiquitous in all compartments of the environment. They are very diverse in nature and are classified into more than 20 different classes [96]. The OPs are classified as industrial chemicals, pesticides, and pharmaceuticals. Polycyclic aromatic hydrocarbons, organochlorine pesticides, polychlorinated dichlorodiphenyltrichloroethane, polybrominated diphenyl ethers, and hexachloro-cyclohexane, are among the most common classes of OPs [97]. These compounds are very toxic; some are mutagenic and carcinogenic even at low concentrations. They are highly stable and may persist in the environment for many decades due to their recalcitrant and hydrophobic nature. These can be absorbed in the food chain and threaten human health, and even at higher concentrations, can cause phytotoxicity. A higher concentration of OPs affects the germination and growth of plants mainly by affecting physiological processes including activities ofkey enzymes, carbon assimilation and several other metabolic events. Like other abiotic stresses, POPs also induce oxidative stress through stimulation of ROS, [98], which leads to lipid peroxidation (LPO) that in turn damage DNA/RNA, and their membranes.

Phytoremediation involves the use of plants for de-contamination of OPs and is a very cost-effective and environment-friendly approach; however, it doesn’t work at higher concentrations of OPs in the environment mainly due to their toxicity in plants. The application of nanomaterials would be effective in reducing the toxicity of OPs to plants. In a study on the effect of Ni/Fe bimetallic-nanoparticles on polybrominated diphenyl ethers (PBDEs) toxicity in Chinese cabbage, nanomaterials significantly decreased the phytotoxicity of PBDEs. This was inferred that the coupling of NMs and bioremediation could reduce the toxicities of soil contaminants and NMs in the plants simultaneously [99]. In another study, the effect of carbon nanotubes (CNTs), on the uptake of four persistent organochlorine insecticides, by lettuce was evaluated. The application of CNTs dramatically decreased the uptake of chlordane components and *p,p*^'^-DDE by lettuce seedlings. The reduced uptake by plants was probably due to the higher sorption capacity of CNTs for OPS [100]. The use of Ag NPs has also been found effective in decreasing the uptake and accumulation of *p,p′*-DDE in Zucchini and soybean [*Glycine max* (L.) [101]. Similarly, the use of multiwalled carbon nanotubes decreased the pyrene and 1-methylpyrene concentrations in roots and shoots, and reduced their translocation in maize [102].

Some NPs such as ZnO, carbon nanotubes, nZVI particles, graphene quantum dots, Ag, etc. could improve plant growth under OPs stress conditions through different mechanisms. For example, ZnO and graphene quantum dots might serve as nanofertilizer [103]. Praveen et al. [104] reported that the application of ZnO NPs would increase tolerance against Fe_2_O_3_ stress in Indian mustard by altering enzyme activities. In addition, NPs may increase growth by improving the uptake and translocation of water and nutrients and by stimulating the soil microbial community.

Biosynthesized nanomaterials have also shown promising results in the degradation of several organic pollutants from the environment. For instance, biosynthesized PdNPs were many folds effective, catalyst powder of commercial palladium, in the dehalogenation of several toxic congeners of polychlorinated biphenyl’s (PCBs) in water and sediments [105]. Likewise, five times more catalytic dehalogenation of a flame retardants tris-(chloroisopropyl)-phosphate (TCPP) was noted with the palletized cells of *Desulfovibrio desulfuricans* compared to the chemically reduced palladium powder [106]. Another study reported that the surface application of biosynthesized PdNPs on a graphite cathode caused a significant increase in the rates of trichloroethylene (TCE) dehalogenation and diatrizoate deiodination [107]. Although several studies have shown the promising results of biosynthesized nanoparticles on the overall dissipation of OPs from the environment, no studies are found on the ameliorative impact of biosynthesized NPs on the phytotoxicity of OPs.

### Hypoxia and anoxia stresses

Due to climate change, plants experience many environmental stresses including submerged and waterlogged conditions [108]. Both stresses cause variable availability of oxygen (O_2_) ranging from normal O_2_ level (normoxia) to hypoxia (partial O_2_ deficiency) and anoxia (total O_2_ deficiency) because O_2_ diffusion is slower in water than in air [15]. Flooding stress/hypoxia conditions inhibit respiration, seed germination, and plant growth [109]. Under O_2_ deficiency (hypoxia and anoxia stresses), the phosphorelation (ATPs generation) significantly reduces. The accumulation of lactate and ethanol, produced during oxygen-deficient respiration, becomes toxic to plants beyond a threshold nd results in cytosolic acidification [110]. Hypoxia-anoxia stresses in plants reduce green pigments, gaseous exchange at stomata, and photosynthetic activity. In addition, hypoxia stress causes oxidative damage by accelerating lipid peroxidation due to the hyperproduction of reactive oxygen species (ROS) in leaf and root tissues [111].

To enhance hypoxia and anoxia tolerance in plants, several molecular and breeding techniques along with natural selection are being used [112]. The exploration and exploitation of novel strategies (nanobiotechnology) complementing existing conventional approaches have become very crucial for sustainable plant growth [112]. The development of green-(bio) nanotechnology by exploiting biological routes helps in minimizing hazards of abiotic stresses in plants including hypoxia/anoxia stresses [113]. In this regard, silicon nanoparticles (SiNPs) -treated plants combat hypoxia stress more efficiently compared with conventional Si by improving antioxidant activities, osmoprotectant accumulation, and micronutrient regulation [111]. Almutairi [114] reviewed that AgNPs and aluminum oxide nanoparticles (Al_2_O_3_NPs) decreased the adverse effects of flooding stress. Similarly, Mustafa et al. [115] used Al_2_O_3_NPs, ZnONPs, and AgNPs in soybeans to alleviate the abiotic stress of flooding. Proteome analysis revealed that most of the protein expressions related to energy metabolism were changed under flooding stress and this change was decreased with the usage of Al_2_O_3_NPs.

Mustafa and Komatsu [116] indicated that the influence of the size of NPs in anoxia tolerance was more prominent in soybean, rather than the quantity and types. Nevertheless, nanomaterials reduce flooding stress (hypoxia and anoxia stresses) and enhance plant growth by hindering ethylene biosynthesis in Arabidopsis [117]. Further studies may be initiated to explore the potential of nanomaterial application in improving plant tolerance against hypoxia and anoxia stresses.

## Nanobiotechnology and tolerance against biotic stresses

The state of a plant in which living organisms (viruses, bacteria, fungi, nematodes, insects, arachnids, and weeds) disrupt their normal metabolic (growth, vigor, and productivity) activities is known as biotic stress. Biotic stresses are responsible to cause significant economic yield losses in major crops like wheat (28.2%), rice (37.4%), maize (31.2%), potatoes (40.3%), soybeans (26.3%), and cotton crops (28.8%) [118]. The weeds alone causes the highest (32%) yield loss followed by animal pests (18%), fungi and bacteria (15%) and viruses (3%) [8]. Thus, a number of pests are responsible for causing infections and ultimately inciting biotic stress to host plants. Iqbal et al. [111] reported that insects and mites impair plants by piercing and sucking the cell sap or chewing their parts. In addition, insects also act as vectors and carriers for different viral and bacterial diseases. Fungi can kill host cells by toxin secretions (necrotrophic) or feed on living host cells (biotrophic). Nematodes cause nutrient deficiency, stunted growth, and wilting by feeding on host plants. Plant pathogenic bacterial infection symptoms include galls and overgrowths, wilts, leaf spots, specks and blights. Similarly, viruses cause damage resulting in chlorosis and stunting the growth of host plants [111]. Plants defend themselves against biotic stresses through their immune system, physical barriers (cuticles, wax, and trichomes) and chemical compounds [119, 120]. Pesticides (herbicides, insecticides, fungicides, etc.) are used as a major component for the management of pests causing biotic stresses [121, 122]. The facts about health hazards and impacts on non-target organisms of pesticides are linked with serious effects on micro and macro flora as well as fauna including negative impacts on human health [26, 123, 124]. In this regard, the use of nanobiotechnology has been proven very effective for the management of the aforementioned issues. Therefore, the potential of nanobiotechnology to counter biotic stress has been highlighted and discussed below.

### Insect pest management

In developing countries, pre-harvest and post-harvest grain crop losses ranging from 15 to 100% and 10–60% [125]. Insects belong to the phylum Arthropoda and 20% of global annual crop losses, valued at over US$ 470 billion are attributed to arthropod pests [126]. In addition to direct damage, insect pests can also transmit or facilitate pathogens (viruses, bacteria, fungi) to cause diseases in plants [127]. The damages reported [128] in major crops due to insect pests are 25%, 5–10%, 30%, 35%, 20%and 50% in rice, wheat, pulses, oilseeds, sugarcane and cotton respectively. The intensive use of pesticides has led to the development of resistance, resurgence and replacement of insect pest species. The resistant insects and mite species had risen to more than 700 [129]. These facts highlight the need for alternate control tactics for the sustainable management of insect pests. The implications of biosynthesized nanoparticles against insect pests have been discussed in this section.

#### Entomopathogenic bacteria with nanoparticles

Bacteria are ubiquitous in nature and have developed a variety of interactions with insects. They have evolved an array of tactics to invade the host insects, and overcome their immune responses to infect and kill them. Entomopathogenic bacteria (EPB) are generally recognized as low-risk substances than conventional synthetic pesticides. The application of EPB alone for pest management has some limitations i.e., (i) ingestion of spores in the host body to cause infection, (ii) solar radiation, (iii) leaf temperature, (iv) vapor pressure, (v) host resistance and can affect the pathogenicity/virulence of these pathogens [130, 131]. Several studies highlight the compatibility and synergistic effects of EPB and nanoparticles for insect pest management [132]. The bioefficacy of EPB-based nanobiopesticides with different biocompatible chemical elements like silver (Ag), zinc oxide (ZnO), copper oxide (CuO, Cu_2_O), gold (Au), etc., have been proven effective (Table 2).

**Table 2:** The influence of nanoparticles on the motality of target insect pests

Type of EPB	Metal	Target Pest	Technical Name	Order/Family	Efficacy	Reference
Entomopathogenic Bacteria + Nanoparticles of different metals						
*Bacillus thuringiensis*	Silver (Ag)	Cabbage looper	*Trichoplusia ni* (Hübner)	Lepidoptera/Noctuidae	Cabbage looper showed dose dependent mortality (40–100%)	[133]
		Black cutworm	*Agrotis ipsilon* (Hufnagel)	Lepidoptera/Noctuidae	Significant mortality (22–83%) of black cutworm was recorded	
		Red palm weevil	*Rhynchophorus* *ferrugineus* (Olivier)	Coleoptera/Curculionidae	Significant larval (85%) and adult (75%) mortality	[134]
	Zinc (Zn)	House Fly	*Musca domestica*	Diptera/Muscidae	Significantredution in LC10, LC20, LC50 and LC90 values of 4.17, 6.11, 12.73 and 38.90 μg/g of larval diet than control	[135]
		Pulse beetle	*Callosobruchus maculatus*	Coleoptera/Chrysomelidae	Caused 100% mortality at 25 μg/mL	[136]
	Ag	Pink Bollworm	*Pectinophora gosypiella*	Lepidoptera/Gelechiidae	Reduced (31.2%) female fertility, prevented the adult emergence and stopped the life cycle	[137]
*Bacillus subtilis*	Ag	Red palm weevil	*Rhynchophorus* *ferrugineus* (Olivier)	Coleoptera/Curculionidae	Significant larval (77%) and adult (67%) mortality	[134]
*Bacillus megaterium*		Dengue vector/ malarial	*Cx. quinquefasciatus* *Ae. Aegypti*	Diptera/Culicidae	Mortality decreases as compared to individual compound	[138]
*Xenorhabdus ssp*	Copper (Cu)	Armyworm	*Spodoptera litura*	Lepidoptera/Noctuidae	80% mortality was observed at 100 µl/mL of biosynthesized CuNPs	[139]
*Xenorhabdus nematophila* NP-1 strain	Ag	Armyworm	*Spodoptera litura*	Lepidoptera/Noctuidae	Significantly highest mortality (90%) after 48 h in 100 µg/mL concentration	[140]
Entomopathogenic Fungi + Nanoparticles of different metals						
*Beauveria bassiana*	Ag	Dengue vector mosquito	*Aedes aegypti*	Diptera/Culicidae	The LC50 and LC 90 values were 0.79 and 1.09 with respect to the *Ae.aegypti* treated with *B.bassiana*, silver nanoparticles. The highest percentage mortality was found 83.3%	[138]
		Mustard aphid	Lipaphis erysimi Kalt	Hemiptera/Aphididae	Silver NPs showed the maximum mortality (60–90%)	[141]
		Mustard aphid	Lipaphis erysimi Kalt	Hemiptera/Aphididae	Isolates B4 and B13 showed the maximum mortality (60.088%)	
		House fly	*Musca domestica*	Diptera/Muscidae	Significant mortility of 1^st^ (95–100%), 2^nd^ (70–100%) and 3^rd^ (60–100%) instar larvae of pest	[142]
		Whitefly	*Bemisia tabaci*	Hemiptera/Aleyrodidae	Green AgNPs of *B. bassiana* JAU2 gave better insecticidal activity causing 8–97% mortality at different concentrations	[143]
	Zn	Greenhouse whitefly	*Trialeurodes vaporariorum*	Aleyrodidae/Hemiptera	Mortality rates obtained with ZnO NPs and fungi at the highest concentration were 91.6% and 88.8%, respectively	[144]
*Metarhizium anisopliae*	Ag		*Anopheles culicifacies*	Diptera/Culicidae	50% mortality of *Anopheles culicifacies* by using silver nanoparticles at 32.8 ppm (I), 39.8 ppm (II), 45.9 ppm (III), 51.9 (IV), and 60.0 ppm (pupa)	[145]
		Red palm weevil	*Rhynchophorus* *ferrugineus* (Olivier)	Coleoptera/Curculionidae	*M. anisopliae* mediated silver nanoparticles caused highest % mortality (90%), (95%) and (77%) against eggs, larvae and adults of *R. ferrugineus*	[134]
*Trichoderma viride*	Titanium (Ti)	American Bollworm	*Helicoverpa* *armigera*	Lepidoptera/Noctuidae	TDNPs exhibited highest mortality rate on first (100%), second (100%) and third (92.34%), instar larvae of *H. armigera* at 100 ppm	[146]
*M. anisopliae*	Ti	Wax moth	*Galleria mellonella*	Lepidoptera/Pyralidae	Prodcued highest mortality percentage (82%)	[147]
*B. bassiana*;*M. anisopliae*; *Verticillium lecanii*;	Ag	Tortoise beetle,	*Cassida vittata*	Coleoptera/Chrysomelidae	3 EPFs caued 47–95% mortality rates within 7 days of exposure	[148]
*Isaria fumosorosea*	zero-valent iron (ZVI)	Whitefly	*Bemisia tabaci*	Homoptera/Aleyrodidae	Mortality increased with increasing concentrations with highest mortality being at 90.12% for 100 ppm	[149]
Sixteen isolates of *B*. *bassiana* (13); *M*. *anisopliae* (2); *I. fumosorosea* (1)	Ag	Mealworm	*Tenebrio molitor*	Coleoptera/Tenebrionidae	B. bassian isolated showed14-94% mortality, *M*. *anisopliae* exhibited 78–86% mortality and *I*. *fumosorosea* caused 10% mortality	[150]
*B*. *bassiana*; *M*. *anisopliae*; *I*.* fumosorosea*		Diamondback moth	*Plutella xylostella*	Lepidoptera/Plutellidae	The CL_50_ value of 0.691 mg/mL was determined at 72-h for the 2^nd^ instar larvae of the *P. xylostella*, causing 78% of cumulative mortality rate	[151]
*M*. *Rileyi*		Armyworm	*Spodoptera litura*	Lepidoptera/Noctuidae	Maximum larval mortality was 80% and 78.75% in laboratory and field conditions	[152]
Plant Extracts + Nanoparticles of different metals						
*Annona muricata*	Ag	Mosquitos	*Aedes aegypti*	Diptera/Culicidae	AgNps exhibited 100% mortality at 48 h observation	[153]
*Euphorbia prostrata*		Rice weevil	*Sitophilus oryzae*	Coleoptera/Curculionidae	71–97% mortality at 50–250 mg/kg concentrations of synthesized silver nanoparticles after 14 days of exposure	[154]
Origanum majorana*		Spotted bollworm	*Earias insulana*	Lepidoptera/Noctuidae	More than 60% reduction of *Earias insulana* infestation	[155]
*Hypnea musciformis*		Diamondback moth	*Plutella xylostella*	Lepidoptera/Plutellidae	LC50 from 24.5 to 38.23 ppm for L1 and pupae of *P. xylostella*	[156]
*Ficus religiosa and* *Ficus benghalensis*		Gram caterpillar	*Helicoverpa armigera*	Lepidoptera/Noctuidae	Significantly reduced both larval weight and survival rate of *H. armigera*	[157]
*Myriostachya wightiana*		Flour beetle	*Tribolium castaneum*	Coleoptera/Tenebrionidae	Moderate significant efficacy against target pests. After 24 h of exposure, at highest concentration (150 µg) biogenic silver treatment was found to be comparatively toxic and killed 55.2% of *T. castaneum*, 52.8 ± 0.24% of *R. dominica* and 47.4 ± 0.16 of *S. oryzae* insects after 24 h	[158]
		Lesser grain borer	*Rhyzopertha dominica* (F.)	Coleoptera/Bostrichidae		
		Rice weevil	*Sitophilus oryzae* (L.)	Coleoptera/Curculionidae		
*Datura stramonium* and *Syzygium aromaticum*		khapra beetle	*Trogoderma granarium*	Coleoptera/Dermestidae	Significanlty highest control 67.89% with biosynthesized nanoparticles	[159]
*Ocimum tenuiflorum*		American bollworm	*Helicoverpa armigera*	Lepidoptera/Noctuidae	50% mortality caused at 0.25% contration of biosynthesized AgNPs causes mortality	[160]
*Vernonia anthelmintica* (L.) Willd		Armyworm	*Spodoptera litura*	Lepidoptera/Noctuidae	86.90% and 89.83% antifeedant activity and larvicidal activity of (LC50) 56.42 μg/mL and 63.65 μg/mL against *S*. *litura* and *H*. *armigera* respectively	[161]
		American bollworm	*Helicoverpa armigera*	Lepidoptera/Noctuidae		
*Borago officinalis*		Cotton leafworm	*Spodoptera littoralis*	Lepidoptera/Noctuidae	LC50 values of the crude extract, and synthesized AgNPs were 22.6 and 0.33 mg/g respectively	[162]
pomegranate and watermelon peels extracts		Cotton leafworm	*Spodoptera littoralis*	Lepidoptera/Noctuidae	additive effect and synergism recorded in results	[163]
*Glochidion eriocarpum*		Termite	*Odontotermes sp*	Isoptera/Termitidae	Strong repellent (80.97%) and antifeedant activity	[164]
*Ocimum basilicum*		Tobacco cutworm	*Spodoptera litura*	Lepidoptera/Noctuidae	ObAgNPs were most effective as compared to the selected synthetic chemicals	[165]
*Nerium oleander*		Common green bottle fly	*Lucilia sericata* M	Diptera/Calliphoridae	100% mortality of treated larvae at 50 ppm	[166]
*Dicrocephala integrifolia*		American bollworm	*Helicoverpa armigera*	Lepidoptera/Noctuidae	Significant Larval mortality and pupal mortality was 68.04% and 72.11% respectively	[161]
		Armyworm	*Spodoptera litura*	Lepidoptera/Noctuidae	Significant Larval mortality and pupal mortality was 69.76% and 74.30% respectively	
*Fusarium pallidoroseum*		White grubs	*Holotrichia sp*	Coleoptera/Scarabaeidae	Lethal dosage (LD50) was significant than control	[167]
*Avicennia marina*	Silver (Ag) + Lead (Pb)	Rice weevil	*Sitophilus oryzae*	Coleoptera/Curculionidae	Aroud 90–100% mortality at 25–100 mg/mL concentration	[168]
*Sargassum wightii*	Zn	American bollworm	*Helicoverpa armigera*	Lepidoptera/Noctuidae	LC50 from 12.278 (larva I) to 20.798 ppm (pupa) and also reduced longevity and fecundity	[169]
*Pongamia pinnata*		Pulse beetle	*Callosobruchus maculatus*	Coleoptera/Chrysomelidae	100% mortality at 25 μg/mL with LC50 to be 10.85 μg/mL	[170]
Spinach leaves		Rice moth	*Corcyra cephalonica* (S.)	Lepidoptera/Pyralidae	Increase in larval mortality, pupal mortality and adult deformity	[171]
*Adhathoda vasica* and *Asafoetida*		American bollworm	*Helicoverpa armigera*	Lepidoptera/Noctuidae	Asafoetida based zinc nanoparticles were More than 80% 2^nd^ instar larval moratliy	[172]
*Eucalyptus globulus* L		Lesser grain borer	*Rhyzopertha dominica* (F.)	Coleoptera/Bostrichidae	LC50 for leaf extract of *E. globulus* and ZnONPs were 1043.06 and 202.11 ppm respectively	[173]
*Zingiber officinale*		Armyworm	*Spodoptera litura*	Lepidoptera/Noctuidae	The 3^rd^ instar larvae of *S*. *litura* and adults of *M*. *euphorbiae* showed 100% mortality 500 ppm	[174]
		Potato aphid	*Macrosiphum euphorbiae*	Hemiptera/Aphididae		
*Punica granatum*	Cu	Green peach aphid	*Myzus persicae*	Hemiptera/Aphididae	There was 40–86% mortality at different concentrations (250–8000 μg/mL)	[175]
*Blumea* *balsamifera* LINN		Fruit Fly	*Bactrocera dorsalis* (HENDEL)	Diptera/Tephritidae	Mortality rates ranged from 25–100% within only 12 h exposure	[176]
Tulasi leaves		Rice moth	*Corcyra cephalonica* (S.)	Lepidoptera/Pyralidae	Increase in larval mortality, pupal mortality and adult deformity	[171]
*Grewia asiatica* L		Termite	*Heterotermes indicola*	Coleoptera/Rhinotermitidae	Significant mortality at 100 ppm	[177]
Rice hust	Silica (SiO_2_)	Rice moth	*Corcyra cephalonica* (S.)	Lepidoptera/Pyralidae	Increase in larval mortality, pupal mortality and adult deformity	[171]
Entomopathogenic Nematodes + Nanoparticles of Different metals						
*Steinernema feltiae* EPNs	Silver (Ag) Gold (Au) Copper (Cu)	Lesser mealworm	*Alphitobius diaperinus*	Coleoptera/Tenebrionidae	Nematodes and nanoparticles caused a high mortality and the extensity of infection in host larvae, from 12 to 100% and from 8 to 83% respectively	[178]
*Steinernema carpocapsae*	ZnO, TiO_2_ and Fe_3_O_4_	Wax moth	*Galleria mellonella*	Lepidoptera/Pyralidae	Survival rate of nematodes decreased with increased concentrations, with no difference between NPs. But both have the significant mortality on wax moth. Nanoparticles had less influence on survival of infective juveniles	[179]

The *Bacillus thuringiensis* kurstaki mediated silver nanoparticles (*Btk*-AgNPs) application against cabbage looper (*Trichoplusia ni* Hübner) and black cutworm (*Agrotis ipsilon* Hufnagel) demonstrated to be significantly more virulent toward larvae of *T. ni* than to *A. ipsilon* [133]. The marine pathogen *Shewanella alga* is known to produce a strong neurotoxin (tetrodotoxin). *Shewanella alga-*mediated AgNPs significantly increased the mortality of 3rd instar white grub beetle, *Lepidiota mansueta* Burmeister (Coleoptera: Scarabaeidae) in all concentrations used [180]. The larvicidal toxicity of *Bt*-AgNPs was significantly higher than control against 3rd instar larvae of *Aedes aegypti* [138]. Similarly, Soni and Prakash [181] reported that *Listeria monocytogenes*, *Bacillus subtilius* and *Streptomyces anulatus*-mediated AgNPs revealed significantly more larval and pupal toxicity against *Culex quinquefasciatus* than *Anopheles stephensi*. In contrast, *An. stephensi* were found more susceptible than *Culex quinquefasciatus* at the adult stage. The *Bacillus megaterium*-mediated AgNPs by using the extracellular method were found to show higher insecticidal efficacy against *Culex quinquefasciatus* and *Aedes aegypti* [138]. Several studies revealed more promising results than control treatments but the focus of research is quite limited (Table 2). Further studies are needed to utilize the potential of EPB-mediated nanobiopesticides for the management of economic insect pests of various crops.

#### Entomopathogenic fungi with nanoparticles

Entomopathogenic fungi (EPF) are natural inhabitants of the soil and are mostly isolated from insect cadavers [182]. The EPF consists of over 100 genera and > 700 species [183, 184]. They provide a direct adaptive response through different mechanisms (adhesion and recognition of host surface), specialized infectious structures (penetrant tubes or appressoria), enzymes (lipase/esterases, catalases, cytochrome P450s, proteases, and chitinases), and secondary metabolites [185]. Currently, there has been a resurgence of interest in EPF use due to increasing insecticide resistance and environmental concerns over pesticide use [123, 186]. Several insect pests of different crops can be managed by EPF [187–189]. The most common fungal infection in fresh water, soil surfaces, and aerospaces environments are *Metarhizum*, *Beauveria*, *Nomurea rileyi*, *Verticillumlecanii*, and *Hirsutella*. The EPF can also produce broad-spectrum secondary metabolites and physical as well as biological alterations to manage insect pests [190].

The formation of nanoparticles utilizing fungus is known as myco-nanotechnology. The nanotechnology integration with EPF for entomotoxicity can enhance their effectiveness many folds (Table 2). The literature revealed the higher pesticidal efficacy of Chitosan Nanoparticle Coated Fungal Metabolite (CNPCFM) than Uncoated Fungal Metabolite (UFM) and Fungal Spores (FS). Chinnaperuma et al. [146] reported a significant reduction of detoxifying enzymes of *Helicoverpa armigera* due to *Trichoderma viride*-mediated biosynthesis of titanium dioxide nanoparticles (TDNPs) and showed significant mortality of 1st instar (100%), 2nd instar (100%) and 3rd instar (92.34%) larvae of *H. armigera*. Similarly, *Sitophilus oryzae* infestation was effectively managed in storage bags treated with nano-based formuations of *B. bassiana* and *M. anisopliae* [191]. The use of the cell filtration method to prepare *B. bassiana* mediated AgNPs showed maximum (60.09%) mortality of *Lipaphis erysimi* [141]. The significant reduction in fecundity of females and malformed development of adults was also reported in potato tuber moth (*Phthorimaea operculella*) when treated with the nano-based formulation of fungus *Metarhizium rileyi* [191].]. The resistance development can also be managed with nano-based EPF formulations. The combination of mycosynthesised TiNPs and *M. anisopliae* revealed synergistic interaction against *Galleria mellonella* larvae with a synergistic factor (SF) of 1.6 for LC50 and 4.2 for LC90 [147]. It was concluded that EPF can be effectively employed for the reduction of increased insect resistance to entomopathogenic fungi. Therefore, nanotechnology integration with EPFs can enhance their efficacy many folds as compared to NPs or EPFs alone.

#### Bonatnicals with nanoparticles

Many plants have biocidal properties and are being used against insect pests due to their efficacy, biodegradability, varied modes of action and low toxicity to non-target organisms [192]. Therefore, many botanical pesticides are used mainly for insect pest management [193–198]. Many studies have listed the plant species with known and yet to be exploited pesticidal properties [199, 200]. The commercially available botanical pesticides sources include *Tanacetum cinerariifolium* (pyrethrum), *Azadirachta indica* (neem), *Schoenocaulon officinale* (sabadilla), *Nicotiana tabacum* (tobacco) and *Ryania speciose* (ryania) [201]. Overall, extract from the leaves, flowers and twigs of many plants can be used as an insecticide [202].

Several promising outcomes of plant extracts integration with nanoparticles of different metals [203–205] are reported. The plant extracts act as capping and reducing agents and convert metals into nanoparticles with the aid of alkaloids, phenolic acids, polyphenols, proteins and terpenoids. The AgNPs and neem extract showed no toxicity against *Cu. quinquefasciatus* and* A*. *aegypti* during individual application of neem extract and AgNO_3_ in aqueous formulations. The neem-mediated AgNPs revealed 0.047 mg/L and 0.006 mg/L LC_50_ values against *Cu. quinquefasciatus* and* A*. *aegypti* [206]. Similar outcomes were reported by [170] in a study of *Pongamia pinnata* leaf extract mediated ZnONPs against pulse beetle. They reported significant variations in hatchability, larval time, pupal period and fertility including 100% mortality of pulse beetle. Similarly, characterization and entomotoxicity of *Hypnea musciformis* mediated AgNPs demonstrated effective management of mosquitos and diamondback moth (*Plutella xylostella* L.) and environment friendly nature [156].

#### Entomopathogenic nematodes with nanoparticles

The phylum Nematoda consists of around 1 million species and only 27,000 species have been described till now [207]. Entomopathogenic nematodes (EPNs) belong to the families Steinemernatidae and Heterorhabditidae. They parasitize soil inhibiting pest insects and kill them due to the associated mutualistic bacteria (Xenorhabdus, Photorhabdus, Heterorhabditis) [208, 209]. EPNs cause infection in individuals of a number of insect orders e.g., Coleoptera, Dictyoptera, Lepidoptera, Hemiptera, and Orthoptera [210].

The EPNs and nanotechnology integration is quite promising and can produce effective control of insect pests (Table 2). However, protocols should be developed to optimize the efficacy of EPNs and nanoparticles as EPNs efficacy is dependent on nanoparticle concentrations and exposure time [211]. For instance, the reproduction rates were enhanced with two concentrations assayed (500 and 1500 ppm) while a little variation was recorded in pathogenicity. Similar results have also been reported in other studies on the mortality of *Steinernema feltiae* (*Owinema biopreparation*) and *Heterorhabditis bacteriophora* (*Nematop biopreparation*) EPNs [212, 213]. The Au nanoparticles also produced similar results of mortality when exposed to *Steinernema feltiae* from Owinema biopreparation of nanoparticles [213]. The mortality increased with increased concentrations of Au nanoparticles. Kucharska et al. [214] used copper (Cu) nanoparticles and also showed *S. feltiae* mortality as well as its ability to control *Alphitobius diaperinus* depending on the exposure time and nano-Cu concentrations. The combined application of EPNs with nanoparticles of different metals (Ag, Au, or Cu) to control lesser mealworm (*Alphitobius diaperinus*) revealed variations of host growth stages sensitivity and susceptibility to *S. feltiae* and *H*. *bacteriophora*. In addition, the negative effect of AuNPs was also recorded on *Alphitobius diaperinus* adults infected by *S. feltiae* (Owinema) [178]. Furthermore, there are also reports of toxicity of nanoparticles (silica, titanium oxide, ZnO, Al_2_O_3_, silver, and Fe_2_O_3_) on *Caenorhabditis elegans* [215–217]. Further research is needed to improve the integration of nanoparticles with EPNs.

### Nanobiotechnology and Management of Plant Diseases

Phytopathogens (fungi, bacteria, mollicutes, nematodes, viruses) causes enormous losses to human society by damaging their food production, economic growth, sustainable agriculture, environmental resilience and natural landscape. In this regard, bacterial diseases are the most damaging and economically significant pathogens invading various agricultural crops. The wide host range, survival capability and sustainable latent infection make bacteria a challenging pathogen to control [218]. Likewise, more than 19,000 fungi are reported to involve in causing diseases in crop plants globally. Additionally, the fungal spores are freely disseminated by wind currents, water, soil, insects, and other invertebrates, which make the whole crop to be invaded [219]. Nevertheless, there is not a single crop that has been free of plant parasitic nematodes (PPNs) infection. They pose a substantial yield loss (~ 173 billion $), annually [220]. Crop rotation is a routine practice to manage PPNs, but the polyphagous characteristics make this tactic unworkable [221]. In contrast, plant viruses have been revealed to cause ~ 50% of total crop losses which is a great threat to worldwide food security [222]. According to one of the surveys, nearly more than 900 plant virus species are responsible to infect over 700 crop species [223]. They are very challenging due to the fact that they are distributed by insect vectors. The virus management relies fundamentally upon (i) immunization, and (ii) prophylaxis measures. Several strategies including chemicals are unable to offer ultimate control [224]. Therefore, a prodigious opportunity prevails to utilize the applications of nanotechnology for the sustainable management of plant disease epidemics.

#### Management of Bacterial Diseases

Bacteria are single-celled prokaryotic organisms exhibiting symbiotic, parasitic, and saprophytic natures. Various factors such as size, density, the shape of NPs, as well as bacterial motility and specificity (gram + ve and − ve) influence the efficacy of nanoparticles [225]. According to previous research work, different concentrations of biosynthesised AgNPs such as 10, 20, 30, and 40 ppm respectively were applied against *Citrus reticulata* suffering from canker disease at different time intervals. The AgNPs at a concentration of 30 ppm were the most effective concentration to produce the resistance in *Citrus reticulata* against canker disease [226]. The direct application of AgNPs eradicates the bacteria responsible for Huanglongbing disease (*Candidatus Liberibacter* asiaticus) on sick trees and reduced 80–90% of bacterial titre [227]. Bacterial leaf blight (BLB) caused by *Xanthomonas oryzae* pv. oryzae (Xoo) were effectively treated with biologically synthesized AgNPs from *Bacillus cereus* strain SZT1 and it was found to be effective weapon for BLB management. The AgNPs significantly increased the plant biomass with a decreased cellular concentration of ROS and increased concentration of antioxidant enzyme activity in the pot-treated plants [228]. Similarly, AuNPs synthesized from biogenic materials exhibited eco-friendly and strong antibacterial properties [229]. Gram-positive and gram-negative bacteria were effectively inhibited by the plant based gold nanoparticles. Furthermore, it has been also reported that biogenic ZnONPs have a much stronger antibacterial impact than chemically generated nanoparticles [230, 231]. Plant extracts such as *O. basilicum T. pratenese, C. fistula* and others have been used for green synthesis of ZnNPs [232]. The antibacterial effect of ZnONPs synthesized by *Olea europaea* on Xoo strain GZ 0003 had an inhibition zone of 2.2 cm at 16.0 µg mL^−1^ that was significantly different from zinc oxide nanoparticles synthesized by *Lycopersicon esculentum* and *Matricaria chamomilla*. The biofilm formation, swimming motility, bacterial cell membrane and bacterial growth of Xoo strain GZ 0003 were significantly affected by ZnONPs [233]. Biosynthesized CuNPs provide significant results because of the antimicrobial ability of these nanoparticles against the bacterial blight of rice [234] and it CuNPs also proved to be less harmful to the environment [235]. The increasing concentrations (50, 100, and 200 ppm) of CuNPs suppressed the bacterial growth by approximately 61%, 64% and 77% compared to the control [236]. Overall, the biologically synthesized nanoparticles have great potential to counter bacterial disease in plants (Table 3).

**Table 3:** The influence of nanoparticles on plant pathogenic bacteria

NPs	Source	Targeted pathogen	Order/ Family	Effects	References
Ag	*Cannabis sativa* extracts	*Pseudomonas syringae* pv. *tomato*	Pseudomonadales/Pseudomonadaceae	Silver nanoparticles containing 90% lower silver content compared to the un-dialyzed silver salt (Ag-UD) exhibited at least 20% more inhibition	[237]
	AgNO_3_ and NaBH_4_	*Ralstonia solanacearum*	Burkholderiales/Burkholderiaceae	Silver nanoparticles formed in EPS1 solution exhibited a concentration-dependent inhibition of bacteria. Silver nanoparticles at 0.8 mg/mL have been shown to have antibacterial activity but a very low cytotoxicity on the RAW264.7 murine macrophage cells	[238]
	*Moringa oleifera*	*Xanthomonas axonopodis* pv citri	Xanthomonadales/Xanthomonadaceae	Biosynthesised AgNPs at different concentrations (10, 20, 30, and 40 ppm) were exogenously applied on the already infected plants (canker) of *Citrus reticulata* at different day intervals. The AgNPs at a concentration of 30 ppm was found to be the most effective concentration against citrus canker	[226]
	*Bacillus cereus* SZT1cereus SZT1	*Xanthomonas oryzae* pv*.* oryzae	Xanthomonadales/Xanthomonadaceae	Silver nanoparticles showed substantial antibacterial potency (24.21 ± 1.01 mm) for *Xanthomonas oryzae* pv*.* Oryzae	[228]
	polyvinylpyrrolidone with metallic silver	*Candidatus liberobacter*	Hyphomicrobiales/Rhizobiaceae	The AgNPs were applied by foliar sprinkling and trunk injection of 93 diseased trees with remarkable results. Both methods produce an 80–90% decrease of bacterial titre	[227]
	Aqueous extract of strawberry waste	*Ralstonia solanacearum*	Burkholderiales/Burkholderiaceae	A strong inhibition zone was found around the paper disc dipped in 100 µg/mL AgNPs, placed in NA media inoculated with *R. solanacearum* and the inhibition zone was absent around the control disc	[239]
	*Dioscorea bulbifera*	*Bacillus sp.*	Bacillales/Bacillaceae	Inhibition zone ranged from 6.00 ± 0.41 to 11.00 ± 0.87 mm was observed at a concentration of 100 ppm	[240]
		*Enterobacter cloacae*	Enterobacterales/Enterobacteriaceae		
	*Penicillium simplicissimum*, *Aspergillus niger*, and *Fusarium oxysporum*	*Pectobacterium carotovorum*	Enterobacterales/Enterobacteriaceae	Inhibition zone was found up to 15.3 mm at a concentration of 100 ppm	[241]
	*Larrea tridentata*	*Clavibacter michiganensis*	Micrococcales/Microbacteriaceae	The disease incidence did not exceed 20%, reduced disease severity by 36%, inhibition of bacterial growth in the tissue (up to 95%)	[242]
	*Eucalyptus globulus*	*Xanthomonas citri pv. Citri*	Xanthomonadales/Xanthomonadaceae	AgNPs and CuNPs in combination showed maximum growth inhibition (21.06 mm) followed by AgNPs (18.26 mm) and CuNPs (15.27 mm)	[243]
Au	*Olea europaea* fruit extract, *Acacia nilotica* and husk extract	*Pseudomonas spp*	Pseudomonadales/Pseudomonadaceae	AuNPs expressed moderate antibacterial activity and inhibition zone up to 8 mm was found	[244]
	*Phoma sp.*	*Xanthomonas oryzae pv. Oryzae*	Xanthomonadales/Xanthomonadaceae	Inhibition rate for sclerotia formation was (15, 33, 74 and 93% at concentrations (10, 20, 40 and 80) μg/mL of AuNPs respectively	[245]
Zn	*Morus alba* plant leaf extract	*Xanthomonas axonopodis pv. Malvacearum*	Xanthomonadales/Xanthomonadaceae	These NPs was found to be very effective in controlling the bacterial spread in comparison to streptomycin that was used as control	[246]
	Sigma-Aldrich, Steinheim	*P. syringae*	Pseudomonadales/Pseudomonadaceae	An inhibition zone of 0.72 mm was observed at concentration of 0.10 mg/mL ZnO NP discs on plates inoculated with *Pectobacterium carotovorum*	[247]
	*Matricaria chamomilla* L., *Olea europaea* and *Lycopersicon esculentum* M	*Xanthomonas oryzae pv. Oryzae*	Xanthomonadales/Xanthomonadaceae	ZnONPs synthesized by *Olea europaeahad* the highest inhibition zone of 2.2 cm at concentration of 16 mg/mL	[233]
	Green tomato extract	*Xanthomonas oryzae* pv. *oryzae*	Xanthomonadales/Xanthomonadaceae	Zinc oxide nanoparticles powder at the concentration of 4.0, 8.0, and 16 μg/mL expressed an inhibitory zone of 2.4, 2.6, and 2.9 cm, compared with that of 1.4, 1.5, and 1.8 cm from bulk zinc oxide, respectively	[248]
	*Matricaria chamomilla*	*Ralstonia solanacearum*	Burkholderiales/Burkholderiaceae	At concentration of 18 µg/mL, Zinc oxide nanoparticles showed the highest inhibition area of 22.3 mm	[249]
	*Bacillus cereus* RNT6	*Burkholderia glumae*	Burkholderiales/Burkholderiaceae	At 50 µg/mL concentration, pathogen growth was reduced by 71.2%	[250]
		*B. gladioli*			
	*Withania coagulans*	*Ralstonia solanacearum*	Burkholderiales/Burkholderiaceae	Highest inhibitory area of 16.2 mm was exhibited at highest concentration (80 μg/mL) of ZnONPs + leaf extract	[251]
Cu	Shell copper and Multivalent copper	*Xanthomonas perforans*	Xanthomonadales/Xanthomonadaceae	Cu-composites significantly decrease disease severity, using 80% less metallic copper in comparison with Cu-mancozeb in field evaluation (P < 0.05)	[252]
	*Datura Innoxia*	*Xanthomonas oryzae* pv. Oryzae	Xanthomonadales/Xanthomonadaceae	CuNPs exhibited effective antibacterial potency against *Xanthomonas oryzae* pv. oryzae with mean inhibition zone of approximately 18 mm	[234]
	*Carica papaya*	*Ralstonia solanacearum*	Burkholderiales/Burkholderiaceae	After an initial incubation (12 h), NPs posed no effect on biofilm. Maximum inhibition (35% and 37%) was observed at dosage of 125 and 250 μg/mL at 24 h and 12% and 38% reduction in biofilms at 72 h respectively	[253]
	*Eucalyptus globulus*	*Xanthomonas citri pv. Citri*	Xanthomonadales/Xanthomonadaceae	AgNPs + CuNPs exhibited maximum inhibitory area of 21.06 mm followed by AgNPs 18.26 mm and CuNPs 15.27 mm	[243]

#### Management of fungal diseases

There are approximately 1.5 million fungal species that are saprophytic and parasitic in nature responsible for ~ 70 to 80% of crop losses equal to 200 billion Euros [254]*.* Myco-nanotechnology presents a greener alternative to chemically generated nanoparticles (Table 4) because of their broad applicability in disease detection and control [255]. Green synthesis of CuNPs utilizing Citron juice (*Citrus medica*) demonstrated repressing effects against the *F. graminearum*, *Fusarium culmorum*, and *F. oxysporum. The F. oxysporum *was shown the most susceptible to CuNPs when compared with *F. graminearum* and *F. oxysporum *[256]. The clove (*S. aromaticum*) bud extract containing CuNPs demonstrated significant antifungal ability against *Aspergillus niger*, *Aspergillus flavus*, and *Penicillium* spp [257]. Similarly, in vitro application of CuNPs against phytopathogens such as *Alternaria alternata*, and *Curvularia lunata*, *Alternaia alternata*, *Phoma destructiva*, *Phytophthora cinnamon*, *Fusarium oxysporum*, *Fusarium solani*, and *Penecillium digitatum* showed fungal growth inhibition at 20 and 60 µg mL^−1^ [258]. An antifungal nanocomposite based on biosynthesized CuONPs was made that has the potential to increase banana roots and seedling growth and also protects them from fungal diseases [259]. The biosynthesis of AuNPs through fresh fruit extract of *P. serrulate* proved more effective against the *Aspergillus flavus*, *Didymella bryoniae*, and *Fusarium oxysporum* compared to traditional fungicides [260]. The uses of *Melia azedarach* leaf extract for the green synthesis of AgNPs against *Verticillium dahliae* in eggplants (*Solanum melongena* L.) both in vitro and in vivo conditions significantly decreased the growth of *Verticillium dahlia* compared with controls [261]. *Trichoderma* spp could produce metal NPs, particularly Ag which is an effective controlling agent against *F. oxysporium* f. sp. ciceri [262, 263]. In addition, AgNPs synthesized by *Trichoderma* spp (*Trichoderma viride*, *Trichoderma hamatum*, *Trichoderma harzianum* and *Trichoderma koningii*) [264] have been used as an antifungal to control four *Fusarium spp* (*F*. *solani*,* F*. *semitectum*,* F*. *oxysporum*, *and F*. *roseum*) which are considered serious soil-borne fungi. The study proved significant inhibitory effects against all four pathogenic Fusarium species [265].

**Table 4:** The influence of nanoparticles on plant pathogenic fungi

NPs	Sources	Targeted Fungi	Order/Family	Effects	References
Ag	*M. azedarach*	*Colletotrichum sp*.	Glomerellales/Glomerellaceae	At 100 mg/ml concentration, Inhibition of radial growth (40.16 ± 2.35) % was observed AgNPs showed a pronounced antifungal potential with EC50 values ranged between 18.4 and 22.8 µg/mL	[266]
		*Fusarium solani*	Hypocreales/Nectriaceae		
		*Alternaria alternate*	Pleosporales/Pleosporaceae		
		*Macrophomina phaseolina*	Botryosphaeriales/Botryosphaeriaceae		
		*R. solani*	Cantharellales/Ceratobasidiaceae		[267]
		*B. cinerea*	Helotiales and Sclerotiniaceae		
		*F. oxysporum*	Hypocreales/Nectriaceae		
		*Acidovorax oryzae*	Burkholderiales/Comamonadaceae		[268]
	*Streptomyces griseoplanus* SAI-25	*Macrophomina phaseolina*	Botryosphaeriales/Botryosphaeriaceae	Highest zone of inhibition (13 mm) was found at concentration of (1000 µg/mL)	[269]
	Rhizospheric microflora of chickpea	*F. oxysporum*	Hypocreales/Nectriaceae	Highest growth inhibition (95%) was found in vitro at dosage of 100 µg/mL	[264]
	*Trichoderma harzianum*	*F. oxysporum*	Hypocreales/Nectriaceae	100% growth inhibition of *F. oxysporum* was observed at 100 ppm	[270]
	*T. harzianum*	*Sclerotinia sclerotiorum*	Helotiales/Sclerotiniaceae	Sclerotia were formed from the precursor sclerotium at the edges of the control plate with a mean of 116.5 ± 7.7 sclerotia. AgNP-TS and AgNP-T plates exhibited no new sclerotia and reduction in mycelial growth	[271]
	*T. harzianum*	*Alternaria alternata*	Pleosporales/Pleosporaceae	Mycelial diameter for A. alternata was reduced 18% at 5 ppm, 42% at 10 ppm and 52% at 20 ppm. Mycelial growth of P. oryzae was reduced 22%, 46% and 68% for each respective nanoparticle’s concentration	[272]
		*Pyricularia oryzae*	Burkholderiales/Comamonadaceae		
		*Sclerotinia sclerotiorum*	Helotiales/Sclerotiniaceae		
	*Streptomyces capillispiralis* Ca-1, and *Streptomyces zaomyceticus* Oc-5	*Alternaria alternata*	Pleosporales/Pleosporaceae	Highest growth inhibition (75%) was observed at concentration of 2 mM	[273]
		*Fusarium oxysporum*	Hypocreales/Nectriaceae		
		*Pythium ultimum*	Peronosporales/Pythiaceae		
		*Aspergillus niger*	Eurotiales/Trichocomaceae		
	Strawberry waste	*Fusarium oxysporum*	Hypocreales/Nectriaceae	The highest concentration (18 µg ml^−1^) of NPs exhibited the inhibition zone of 22.3 mm	[249]
	*Melia azedarach*	*F. oxysporum*	Hypocreales/Nectriaceae	Growth inhibition (79–98%) was observed	[274]
	*Pseudomonas aeruginosa*	*Botrytis cinerea*	Helotiales/Sclerotiniaceae	At the concentration of 100 ppm, maximum growth inhibition was observed up to 65.36%	[275]
		*Pilidium concavum*	Helotiales/Leotiomycetidae		
		*Pestalotia sp*	Xylariales/Amphisphaeriaceae		
	*T. harzianum* Th3	*Aspergillus niger*	Eurotiales/Trichocomaceae	Mycelial growth inhibited up to 60–65%	[276]
		*Sclerotium rolfsii*	Atheliales/Atheliaceae		
		*Macrophomina phaseolina*	Botryosphaeriales/Botryosphaeriaceae		
	*Dioscorea bulbifera*	*F. oxysporum*	Hypocreales/Nectriaceae	Highest inhibition (98–100%) was expressed at concentration of 100 ppm	[240]
		Colletotrichum gloeosporioides	Incertae sedis/ Glomerellaceae		
	*Bacillus subtilis*	*Cercospora canescens*	Capnodiales/Mycosphaerellaceae	The highest mycelial inhibition (94.00 ± 0.5) was observed at dosage of 800 ppm at 96 h	[277]
	*Bamboo leaf extract*	*Bipolaris maydis*	Pleosporales/Pleosporaceae	Complete inhibition of conidia germination (100%) was detected at concentration of 100 μg/mL	[278]
		*Exserohilum turcicum*	Pleosporales/Pleosporaceae		
		*Curvularia lunata*	Pleosporales/Pleosporaceae		
Au	*Glechoma hederacea* *L.* extract	*Fusarium oxysporum*	Hypocreales/Nectriaceae	Zone of inhibition exhibited by AuNPs against tested pathogens ranges from 30 to 66%and 40 to 54% respectively	[279]
		*Aspergillus niger*	Eurotiales/Trichocomaceae		[280]
	*Annona muricata*	*Aspergillus flaws*	Eurotiales/Trichocomaceae	Zone of inhibition (30—66%) was observed	[279]
		*Fusarium oxysperium*	Hypocreales/Nectriaceae		
	*Phoma sp.*	*Rhizoctonia solani* AG1-IA	Cantharellales/Ceratobasidiaceae	Growth inhibition (93%) was found at concentration of 80 μg/mL	[245]
Zn	*Aloe vera* extract	*Alternaria alternata*,	Pleosporales/Pleosporaceae	ZnNPs at both concentrations of 2 mg/L and 4 mg/L expressed inhibition zone of 50% and 54% respectively	[281]
		*Aspergillus niger*	Eurotiales/Trichocomaceae		
		*Botrytis cinerea*	Helotiales/Sclerotiniaceae		
		*Fusarium oxysporum*	Hypocreales/Nectriaceae		
		*Alternaria mali*	Pleosporales/Pleosporaceae		
		*Botryosphaeria dothidea*	Botryosphaeriales/Botryosphaeriaceae		[282]
		*Diplodia seriata*	Botryosphaeriales/Botryosphaeriaceae		
	*Scadoxus multiflorus*	*Aspergillus niger and Aspergillus flavus*	Eurotiales/Trichocomaceae	Zinc oxide nanoparticles expressed significant antifungal potency against *A. flavus*, with 75% inhibition at 500 ppm and 76% inhibition at 1000 ppm, while *A. niger* resulted in 57% and 63% growth reduction, respectively	[283]
	*Morus nigra and Grevillea robusta*	*Cercospora beticola*	Capnodiales/Mycosphaerellaceae	Nanoparticles led to activation and recorded high POD value up to 6 min and polyphenoloxidase up to 4 min estimation periods compared to control and expressed as role in defense against CLS disease	[284]
	Strawberry Plants	*Botrytis cinerea*	Helotiales/Sclerotiniaceae	The most effective concentrations were 26 and 42 mg/ml for non-calcinated and calcinated zinc oxide nanoparticles, respectively. Inhibition in the fungal growth enhanced with the increase in the concentration of NPs	[285]
	*Sargassum vulgare*	*Aspergillus sp.*	Eurotiales/Trichocomaceae	At concentration of 25 μg/mL, 25 mm inhibition zone was found	[286]
		*Candida sp.*	Hyphomicrobiales/Rhizobiaceae		
		*Saccharomyces cerevisiae*	Saccharomycetales/Saccharomycetaceae		
	*Trichoderma harzianum*	*Alternaria alternata*	Pleosporales/Pleosporaceae	Significantly reduced the mycelial growth (20 mm inhibition zone)	[272]
		*Pyricularia oryzae*	Burkholderiales/Comamonadaceae		
		*Sclerotinia sclerotiorum*	Helotiales/Sclerotiniaceae		
	Seed coat of almond	*Rhizoctonia solani*	Cantharellales/Ceratobasidiaceae	Reduction of pathogen growth up to 100%	[287]
Cu	Magnolia leaf extract	*Botryosphaeria dothidea*	Botryosphaeriales/Botryosphaeriaceae	Mycelial growth inhbited up to 22 mm	[288]
		*Diplodia seriata*	Botryosphaeriales/Botryosphaeriaceae		
		*Colletotrichum gloeosporioides*	Glomerellales/Glomerellaceae		
		*Colletotrichum lindemuthianum*	Glomerellales/Glomerellaceae		
		*Drechslera sorghicola*	Pleosporales/Pleosporaceae		
		*Fusarium oxysporum f.sp. carthami*	Hypocreales/Nectriaceae		
		*Fusarium oxysporum f.sp. cicero*	Hypocreales/Nectriaceae		
		*Fusarium oxysporum f.sp.*	Hypocreales/ Nectriaceae		
	*Citrus sinesis*	*Colletotrichum* *Capsici*	Glomerellales/Glomerellaceae	Maximum antifungal activity (28.00 ± 081 mm diameter) was observed at dosage of 200 ppm	[289]
	*Azadirachta indica*	*Alternaria mali*	Pleosporales/Pleosporaceae	More than 40% of mycelial growth inhibition was found at 1 g/mL of all the fractions	[290]
		*Diplodia seriata*	Botryosphaeriales/Botryosphaeriaceae		
		*Botryosphaeria dothidea*	Botryosphaeriales/Botryosphaeriaceae		
	*Trichoderma harzianum*	*Alternaria alternata*	Pleosporales/Pleosporaceae	Mycelial diameter for *A. alternata* was reduced 18% at 5 ppm, 42% at 10 ppm and 52% at 20 ppm	[272]
		*Pyricularia oryzae*	Burkholderiales/Comamonadaceae		
		*Sclerotinia sclerotiorum*	Helotiales/Sclerotiniaceae		
	*Aspergillus flavus*	*Aspergillus niger*	Eurotiales/Trichocomaceae	Growth inhibition was reduced by 19% at 6 ppm, 40% at 12 ppm and 55% at 20 ppm	[291]
		*Fusariumoxy sporum*	Hypocreales/ Nectriaceae		
		*Alternaria alternata*	Pleosporales/Pleosporaceae		
	*Kappaphycus alvarezii*	*Colletotrichum* *Capsici*	Glomerellales/Glomerellaceae	Complete growth inhibition observed at concentration of 100 ppm	[292]
	*Macrophomina phaseolina*	*Fusarium verticillioides*	Hypocreales/ Nectriaceae	100% growth inhibition observed at concentration of 50 ppm	[293]
		*Sclerotium rolfsii*	Atheliales/Atheliaceae		
	*Pseudomonas fluorescens* and *Trichoderma viride*	*Phytophthora parasitica*	Peronosporales/Peronosporaceae	Maximum percent inhibition ( 74.8%) was observed at 150 mg/L	[294]
	*Grewia asiatica* L	*Aspergillus niger* and *Aspergillus oryzae*	Eurotiales/Trichocomaceae	Significant inhibition was recorded at 20 mm and 23 mm due to Maximum	[177]

#### Management of viral diseases

Nanotechnologies based on biologically synthesize nanoscale materials offer great potential to use as a novel and eco-friendly antiviral therapy for plant disease management (Table 5). For instance, a recent study used ginger and horsemint extracts for biosynthesis of ZnNPs and reported an increase in viral suppression and inhibition [295]. Likewise, Zn and ammonium synthesized NPs from spearmint and plant flavanol extracts showed strong antiviral activity against tomato mosaic virus (TMV). Another work revealed antiviral activity against cucumber mosaic virus (CMV) by using seaweed extract-mediated ZnNPs [296]. The outcome of this study suggested that ZnNPs could serve as a strong antiviral agent due to their promising antiviral characteristics. A similar observation was also witnessed in the work of El-Shazly et al. [297], who synthesized the AgNPs from salicylic acid (SA) and investigated the strong antiviral activity to counter potato virus Y (PVY). It was found that biosynthesized NPs may enter the viral cell and start their antiviral mechanism via interacting with viral genetic material (RNA or DNA) or by preventing the channels that are indispensable for viral reproduction. Different entomopathogenic bacteria were also used to synthesize AgNPs and exploited them for antiviral activity on Tobacco mosaic virus (TMV), Barley yellow mosaic virus (BaYMV), Sunhemp rosette virus (SHRV) and Bean yellow mosaic virus (BYMV) [298–301]. After a post-infection treatment of a certain incubation time as required for each virus, it was observed that all plants showed typical symptoms of TMV, BaYMV, SHRV and BYMV infection. However, plants treated with NPs exhibited negative symptoms of virus infection. Moreover, viral concentration and disease severity were also observed very low in synthesized NPs treated plants (Table 5). Chitosan is another nontoxic biodegradable compound consisting of different monocrotaline and pyrrolizidine alkaloids and has a strong antiviral activity against virus replication and severity of tobacco mosaic virus (TMV) and alfalfa mosaic virus (AMV) [302, 303]. The biologically (plant extract or pathogen based) synthesized NPs are capable of inhibiting plant virus replication and improving host plant growth, however further studies are needed to identify different biological sources for NPs synthesis which are nontoxic to human health.

**Table 5:** The influence of nanoparticles on plant viral and nematode disease suppression

Type of EPB	Metal	Target Virus	Genome	Order/Family	Efficacy	Reference
Virus section						
Different plant extracts + Nanoparticles of different metals						
Spearmint	ZnNPs	TMV	+ ssRNA	*Virgaviridae*	Strong antiviral activity at a concentration of ca ~ 50 ppm	[304]
Ginger Horsemint	SiNPs	TYLCV	ssDNA	*Geminiviradae*	Suppression of TYLV infection at 100 μg/ml of PPE-AuNPs	[295]
Plant flavonol	AuNPs	TMV	+ ssRNA	*Virgaviridae*	Enhanced Antiviral activity and limit the traditional chemicals applications when plants treated with Rugby 60% (4 mL/L)	[305]
Salicylic acid Seaweed	AgNPs ZnNPs	PVY CMV	+ ssRNA	*PotyviridaeBromoviridae*	Decrease viral infection and increase in growth and yield at 7.89 μg/ml (LC50)	[297]
Entomopathogenic/bacteria/viruses + Nanoparticles of different metals						
Pseudomonas fluorescens	AgNPs	TMV	+ ssRNA	*Virgaviridae*	Antiviral activity at 100 μl AgNPs	[306]
Bacteriophage	AgNPs AuNPs	BaYMV	+ ssRNA	*Potyviridae*	Enhance resistance against BaYMV 100–120 μl AgNPs/AuNPs	[307]
Bacillus thuringiensis	AgNPs	SHRV	+ ssRNA	*Virgaviridae*	Complete viral disease suppression at 20 μg per pot	[308]
Bacillus licheniformis	AgNPs	BYMV	+ ssRNA	*Potyviridae*	Antiviral activity at (1: 0.5 v/v)	[300]
Chitosan	Dextran nanoparticles (D-NPs	AMV	+ ssRNA	*Bromoviridae*	Strong elicitor for viral disease control at 100 µg/mL	[303]
chitosan Schiff	AgNPs	TMV	+ ssRNA	*Virgaviridae*	Inhibit virus replication and severity at 100 µg/ml application	[302]
Virus-Capsid	AgNPs	CPMV	+ ssRNA	*Comoviridae*	Virus control at 75 mg/L	[309]
Recombinase	AuNPs	TYLCV	ssDNA	*Geminiviradae*	Highly sensitive in virus detection and diagnosis at 100 µg/mL foliar application	[310]
dsRNA-Virus	Chitosan	ToMV	+ ssRNA	*Virgaviridae*	Decrease toxicity at 17 mg/mL	[311]

Nematode Section						
Different plant extracts + Nanoparticles of different metals						
Type of EPB	Metal	Target Nematode	Family	Order	Efficacy	Reference
Pomegranate	AuNPs	*C. elegans*	*Rhabditidae*	Rhabditida	Adversely affect the fertility of *C. elegans* at 100 μg/mL	[312]
Bermuda grass	AgNPs	*M. incognita*	*Heteroderidae*	Tylenchida	Nematode production was limited at 17 mg/mL	[313]
African locust bean	AuNPs	*B. xylophilus*	*Parasitaphelenchidae*	Aphelenchida	Inhibited the growth of nematodes after 72 h, the mortality range ranged from 87.00% to 98.50% at 150 μg/mL	[314]
Jacob’s coat	AgNPs	*M. incognita*	*Heteroderidae*	Tylenchida	Reduction in nematode population and retard movement of larvae at 21.70 µg/mL	[315]
Strawberry	AgNPs	*M. incognita*	*Heteroderidae*	Tylenchida	Antinematicidal effect at 1–100 μg/L	[239]
Ginger	AgNPs	*M. incognita*	*Heteroderidae*	Tylenchida	Restrict nematode infection at EC_50_ = 3.7 μM and improved plant growth	[316]
Drumstick tree	AgNPs	*M. incognita*	*Heteroderidae*	Tylenchida	Reduce number of nematodes and their eggs at 300 ul and 400 ul concentrations	[317]
Cassod tree	AgNPs	*M. incognita*	*Heteroderidae*	Tylenchida	Have nematicidal activity and is cost-effective at 7.89 μg/mL (LC50)	[318]
Geen Algae	AgNPs	*M*. *javanica*	*Heteroderidae*		Strong nematicides at 4 mL/L	[319]
Neem	AgNPs	*H*. *contortus*	*Trichostrongylidae*		Exhibited potent anthelmintic properties at LC_50_ (588.54 μg/mL)	[320]
Cnidoscolus aconitifolius	AgNPs	*M*. *incognita*	*Heteroderidae*	Tylenchida	Limit *M. incognita* number at 5 mL/L and enhance host plant growth	[321]
Lemon grass	FeO-NPs	*C*. *elegans*	*Rhabditidae*	Rhabditida	Effectively control the *C. elegans* population at 1–100 μg/L	[322]
Fresh water *Cladophora glomerata* and marine alga *Ulva fasciata*	AgNPs	*M*.* incognita*	Heteroderidae	Tylenchida	Significant egg hatching and J2 mortality (up to 100%) compared with Nemaphose (40%) at 17 mg/mL	[323]
Eucalyptus	NPs	*M*.* incognita*	*Heteroderidae*	Tylenchida	Reduce nematode population at 4–6 mL/L and increased vegetative growth of host plant	[324]
Neem Turmeric	NPs	*M. incognita*	*Heteroderidae*	Tylenchida	Significant mortality of J2-nematode at 70–100 µg/mL	[325]
Common mallow	AgNPs	*M. javanica*	*Heteroderidae*	Tylenchida	Antinematicidal effect at 100 µg/mL	[326]
Pencil cactus	AgNPs	*M. incognita*	*Heteroderidae*	Tylenchida	Infestation of *M. incognita* significantly reduced after exposure at (100 ppm) concentration	[327]
Chinaberry Drumstick tree	AgNPs	*M*. *incognita*	Heteroderidae	Tylenchida	Strong nematicidal activity against *M. incognita* at 25 μg/mL	[328]
*Colpomenia sinuosa* and *Corallina mediterranea*	AgNPs	*M*.* incognita*	Heteroderidae	Tylenchida	Significant mortality (87.5%) after 12 h and 100% after 24 and 72 with 100 and 200 ppm	[329]
*Ulva fasciata*	ZnNPs + oxamyl	*M*.* incognita*	Heteroderidae	Tylenchida	Significant diminution of J2s (82.77%) in soil and galls number (81.87%) in vivo conditions	[330]
*Orobanche aegyptiaca*	Cu	*M*. *incognita*	*Heteroderidae*	Tylenchida	Significantly enhanced nematicidal activity at 50 μg/mL	[331]
Entomopathogenic fungus + Nanoparticles of different metals						
Sea lettuce brown algae	AgNPs				Effectively controlled nematodes at 17 mg/mL	[332]
Fusarium oxysporum	SiNPs	*M. incognita*	*Heteroderidae*	Tylenchida	Limit nematode reproduction, gall formation and egg masses with 100 and 200 ppm	[333]
Duddingtonia flagrans	AgNPs	*A. caninum*	*Ancylostomatidae*		Showed nematicides activity, penetrate into the cuticle of the larvae and kill the nematode LC50 (25 μg/mL)	[334]
Neuronal Gα- protein	PS-NPs	*C. elegans*	*Rhabditidae*		Control neuron response of the organism at 1–100 μg/L	[335]
Virus-Capsid	TMGMV-NPs	*M*. *incognita*	*Heteroderidae*	Tylenchida	control parasitic nematode at EC_50_ = 13.8 μM	[336]
Bacteria + Nanoparticles of different metals						
*Bacillus licheniformis* strain GPI-2	AuNPs	*M*. *incognita*	*Heteroderidae*	Tylenchida	100% mortality at EC_50_ = 3.7 μM	[337]

#### Management of nematode diseases

Plant parasitic nematodes (PPNs) are holoparasites which pose a substantial yield loss (~ 173 billion $) annually [220]. Around 4100 species of PPNs have been recognized, and a majority of them are polyphagous [338]. Biologically (plant, fungus, or bacteria) synthesized NPs exhibiting a strong nematicide activity [309]. For instance, AuNPs synthesized by pomegranate peel and African locust bean extracts, effectively inhibit the multiplication and reduced the fertility of Caenorhabditis elegans and *Parasitaph elenchidae* [255, 312, 339]. Likewise, biologically synthesized AgNPs by using more than 14 plants showed significant mortality of J2 nematode, and antifilarial effects against *Meloidogyne incognita*, *M. javanica*, *H. contortus*, *C*. *elegans* and *Setaria cervi* [313, 318, 320, 323–325, 327, 340–346]. The presence of flavonoids and other phenolic compounds in plant extracts may help improve the efficiency of the NPs in inhibiting nematode populations [347]. The strong effect of AgNPs might be due to the high concentration of secondary metabolites including epi-shyobunol, aromdendrene, α- and t-cadinol, caryophyllene, α-humulone, β-isocomene, and α- and β-selinene) [328]. The aqueous leaf extract of Jacob’s coat and strawberry waste extract and their antinematod activity restricts the movement of *M. incognita* but have the ability in preventing both, eggs and J2 stages of nematode population [239, 342]. Different polymers and compounds were investigated from various entomopathogenic fungi including Fusarium oxysporum, Duddingtonia flagrans, sea lettuce (Ulva lactuca), and brown algae to synthesize AgNPs, SiNPs, ZeinNPs, AgB and their nematicidal activity was successfully evaluated [333, 334, 348]. The findings verified the successful inhibition and reduction of J2 population of nematodes. The growth of plants was also remarkable. It was witnessed from different research findings that biologically synthesized NPs exhibited strong nematicidal activities (Table 5).

## Challenges/risks of nano biotechnology

Nanobiotechnology could be an important driver for the imminent agri-tech revolutions, especially in the face of climate change and increasing populations which make the existing agricultural practices unsustainable. Therefore, it is needful to explore more about nanomaterials and their characteristics as they behave totally differently than in bulk form. There are concerns that some materials could be toxic at the nanoscale because of their significant, still mysterious, hazardous properties related to their unique physico-chemical characteristics. This can pose risks for a wide range of manufacturers, formulators, handlers, applicators and also the consumers. Therefore, nanotechnology causes heterogeneous effects [349]. Toxic effects on non-target organisms upon contact i.e., nanoparticles can come in direct contact with humans and can cause unfavorable or undesirable toxic effects on humans. The nanoparticles can reach various human body parts to exert ill effects and may disrupt cellular pathways, enzymatic actions and functions of different organs. The disposal of nanomaterials might form a new class of non-biodegradable pollutants in the environment. Nanomaterials can enhance environmental pollution by increasing water, soil and air contamination including health hazards. Studies on nanotoxicity in agriculture are limited but reveal potential risks to plants, beneficial microbes, animals, and even humans. Health issues of workers during different activities (production, packaging, formulations, loading, unloading, or transport) of nanomaterials. The hazardous effects of nanoparticles on non-target organisms include dermal absorption of nanoparticles, translocation to go deep into lungs and brain through inhalation and crossing the brain barriers respectively, environmental concerns due to resilience and reactive potential of some nanomaterials, and lack of knowledge to estimate environmental exposure can pose a number of risks to different stakeholders including human beings.

The possible risks associated with nanomaterials lead to the challenges of basic as well as applied nanobiotechnology in different sectors including crop production. The major possible challenges [349] are:

(i) Mass production of nano-based products with standard quality at an economical cost.
(ii) Availability of nanomaterials in ready to use product with proper particle size, surface chemistry, etc.
(iii) The establishment of a customized nanomaterial production system to fulfill local needs.
(iv) Environmental and human health safety and protection during the use and disposal of nanomaterials.
(v) The challenge to overcome the gap between basic and applied nano-based research.
(vi) The cost of production, intensive risks, and technical knowledge gaps are also considered as major concern/challenge in nanobiotechnology applications.
(vii) The problems faced by regulatory institutions and the lack of inter-institution coordination are the main challenges in the current situations of applied nanobiotechnology.

Therefore, governmental and workforce efforts based on sound scientific research, and technological advancements should be focused to meet the described challenges and associated risks. This would provide the necessary information to devise appropriate guidelines for comprehensive risk management and applications of nanobiotechnology for sustainable crop production.

## Conclusion/future perspectives

The biologically synthesized nanoparticles provide promising solutions against biotic (insect pests, plant diseases) and abiotic (drought, salinity, thermal stresses, toxic metals, organic pollutants) stress factors. Nonetheless, nanobiotechnology application in the agriculture sector is at its nascent stage to counter biotic and abiotic stress factors. Remarkable work has been done regarding biosynthesized nano-based formulations but most of the work has been done in vitro conditions while in vivo applications are lacking. Therefore, only a few green nanotechnology-based products are available in the market may be due to production cost, a major hindrance along with other environmental issues towards wider marketing which could be overcome by promoting green nanoformulations. that the industrial scale manufacturing of green nanomaterials, not yet been widely started, can help with affordable prices, and is safer due to little chemical usage and low energy requirements in nanobiotechnology. The biologically synthesized nanoparticles also have some limitations regarding their stability and degradability in the environment which need to be addressed through innovative techniques of application and integration with other molecules. Therefore, it is important to understand plant–nanoparticle interaction and optimization of size, concentration and compatibility of NPs with biological systems before practical applications in the fields to reduce the degradability and negative impact on the natural environment and crops as well. Conclusively, nanobiotechnology requires comprehensive basic and advanced research on fabrication, characterization, standardization, biodegradability and also possible uptake and translocation of nanoparticles by plant systems for sustainable crop production and protection from biotic and abiotic stress factors.

## Data Availability

All data generated or analyzed in this study are included in this article. The authors also declare that they have no conflict of interest.

## Code availability

No codes were used in the manuscript.
