# Correction: Nanobiotechnology in crop stress management: an overview of novel applications

**DOI:** 10.1186/s11671-023-03876-8

**Published:** 2023-08-21

**Authors:** Ahmad Nawaz, Hafeez ur Rehman, Muhammad Usman, Abdul Wakeel, Muhammad Shafiq Shahid, Sardar Alam, Muhammad Sanaullah, Muhammad Atiq, Muhammad Farooq

**Affiliations:** 1 Department of Entomology, University of Agriculture, Faisalabad, 38040 Pakistan; 2 Department of Agronomy, University of Agriculture, Faisalabad, 38040 Pakistan; 3 PEIE Research Chair for the Development of Industrial Estates and Free Zones, Center for Environmental Studies and Research, Sultan Qaboos University, Al-Khoud 123, Muscat, Oman; 4 Institute of Soil and Environmental Sciences, University of Agriculture, Faisalabad, 38040 Pakistan; 5 Department of Plant Pathology, University of Agriculture, Faisalabad, 38040 Pakistan; 6 Department of Plant Sciences, College of Agricultural and Marine Sciences, Sultan Qaboos University, Al-Khoud 123, Muscat, Oman

**Correction to: Discover Nano (2023) 18:74** **https://doi.org/10.1186/s11671-023-03845-1**

The original article [1] contained an error in the affiliation of author Muhammad Shafiq Shahid:

The author is affiliated with the Department of Plant Sciences, College of Agricultural and Marine Sciences, Sultan Qaboos University, and not with the Department of Plant Pathology, University of Agriculture, Faisalabad 38,040, Pakistan, as previously indicated in the article.

The correct affiliation is shown in the 'Author details' of this Correction, as well as in the original article, which has now been updated.

